# Beyond the income‐achievement gap: The role of individual, family, and environmental factors in cognitive resilience among low‐income youth

**DOI:** 10.1002/jcv2.12297

**Published:** 2024-12-20

**Authors:** Divyangana Rakesh, Ekaterina Sadikova, Katie A. McLaughlin

**Affiliations:** ^1^ Neuroimaging Department Institute of Psychiatry Psychology & Neuroscience King's College London London UK; ^2^ Department of Psychology Harvard University Cambridge Massachusetts USA; ^3^ Department of Social and Behavioral Sciences Harvard T.H. Chan School of Public Health Boston Massachusetts USA; ^4^ Ballmer Institute University of Oregon Eugene Oregon USA

**Keywords:** ABCD study, childhood and adolescence, cognitive function, poverty, resilience, socioeconomic status

## Abstract

**Background:**

Low socioeconomic status is associated with lower cognitive performance and long‐term disparities in achievement and success. However, not all children from low‐income backgrounds exhibit lower cognitive performance. Characterizing the factors that promote such resilience in youth from low‐income households is of crucial importance.

**Methods:**

We used baseline data from participants in the lowest tertile of income‐to‐needs in the Adolescent Brain Cognitive Development study and machine learning to identify the factors that predict fluid and crystallized cognitive resilience among youth from low‐income backgrounds. Predictors included 164 variables across child characteristics, family and developmental history, and environment.

**Results:**

Our models were reliably able to predict resilience but were substantially more accurate for crystallized cognition (AUC = 0.75) than for fluid cognition (AUC = 0.67). Key predictors included developmental factors such as birthweight and duration of breastfeeding, neighborhood‐level factors (e.g., living in concentrated privilege, enrollment in advanced placement courses), children's own temperament and mental health, and other factors such as physical activity and involvement in extracurricular activities.

**Conclusion:**

Our findings highlight the importance of a multifaceted approach to promoting cognitive resilience among children from low‐income households in future intervention work.

## Graphical abstract

Machine learning based prediction of cognitive resilience among low income youth.

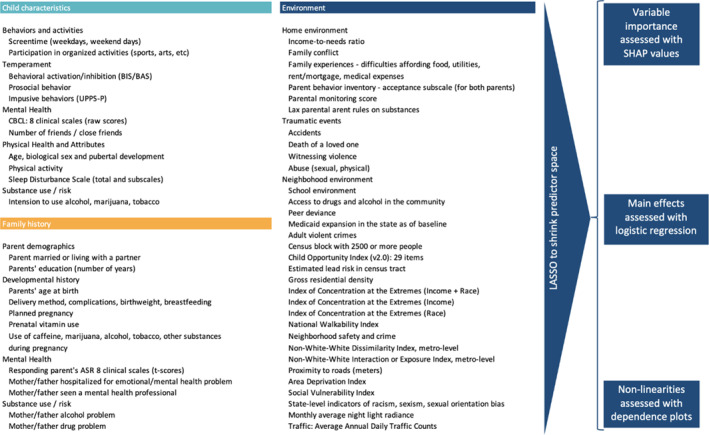

**Key points**

**What's known?**

- Not all children from low‐income backgrounds exhibit lower cognitive performance.
- The factors that promote such resilience in youth from low‐income households remain unknown.

**What's new?**

- We used machine learning to identify the factors (including child characteristics, family and developmental history, and environment) that predict fluid and crystallized cognitive resilience among youth from low‐income backgrounds.

**What's relevant?**

- We found developmental factors such as birthweight and duration of breastfeeding, neighborhood‐level factors (e.g., living in concentrated privilege, enrollment in advanced placement courses), children's own temperament and mental health, and other factors such as physical activity and involvement in extracurricular activities to predict cognitive resilience.
- Our findings highlight the importance of a multifaceted approach to promoting cognitive resilience among children from low‐income households in future intervention work.

## INTRODUCTION

Low socioeconomic status (SES) is associated with lower cognitive performance (Lawson et al., 2018; Noble et al., 2007) creating long‐term disparities in achievement and success (Best et al., 2011). Despite considerable efforts, the SES‐achievement gap has persisted globally (Chmielewski, 2019). Beyond individual wellbeing and success, childhood poverty costs the USA government >$1 trillion a year in part due to loss of economic productivity (McLaughlin & Rank, 2018). Importantly, not all children from low‐income backgrounds exhibit lower cognitive performance. However, most studies have adopted a deficit‐based approach, which overlooks this heterogeneity present within youth living in poverty (DeJoseph et al., 2024). Adopting an adaptation or strength‐based framework and characterizing the factors that promote positive outcomes in youth from low‐income households is essential to inform targeted interventions aimed at alleviating the negative sequalae of poverty.

Although growing up in poverty carries many risks, some children defy the odds and demonstrate positive outcomes. These children are regarded as resilient (Sattler & Gershoff, 2019). Although resilience is a widely studied concept in developmental science, particularly in the context of mental health, consensus on how to define the construct is still lacking (Masten, 2014). Widely used definitions describe resilience as “doing better than others facing similar risks” (Rutter, 2006) or doing as well peers not facing the same risks (Luthar et al., 2000), both of which require the setting of a threshold to classify individuals as resilient versus not, a common approach adopted in developmental studies (Luthar et al., 2000; Luthar & Cicchetti, 2000; and see studies included in Zhang et al., 2023). Importantly however, only “high threshold resilience” (i.e., performing as well as peers not facing the same risks) has been shown to be associated with improved long‐term outcomes (Sattler & Gershoff, 2019). Another definition considers resilience as the adaptive “capacity of a dynamic system” and focuses on the interplay between risk and protective factors that may promote positive outcomes in the context of adversity (Masten, 2014). Our study combines these definitions to characterize the factors that promote high cognitive function among children from low‐income backgrounds.

While numerous studies have investigated positive outcomes despite adversity in the context of mental health and academic achievement, studies on cognitive performance are less common among adolescents. While cognitive performance is related to academic achievement, they are only moderately correlated (Tikhomirova et al., 2020), indicating that they are distinct constructs. Indeed, executive function—including working memory, flexibility, and attention—has consistently been shown to mediate associations between SES and academic achievement (Rakesh et al., 2024) suggesting that it lies earlier in the causal chain linking low SES with lower achievement. Furthermore, academic achievement reflects socio‐emotional skills, and external factors like educational support from family and teachers (Gruijters et al., 2024). While several studies have focused on cognitive resilience in older adults and clinical populations (e.g., Graham et al., 2021; Willroth et al., 2023), research on cognitive resilience among children, particularly those from low‐income backgrounds is limited. Therefore, studies that examine factors that may promote higher cognitive function in children from low‐income backgrounds are warranted.

Numerous studies have examined individual‐level factors that contribute to positive cognitive and academic outcomes in youth. However, cognitive development and academic achievement are influenced by a range of factors operating at not only the individual but also the household, community, and school levels. For instance, at the individual level, children's pubertal development has been linked to executive function (Stumper et al., 2020), and temperamental reactivity has been shown to reduce the strength of the association between low SES and lower executive function (Raver et al., 2013). Children's behavior and activities, such as sleep duration, screen time, and participation in extracurricular activities are also associated with cognitive function (Kirlic et al., 2021).

Developmental and family history also play a role in children's cognitive outcomes. For example, birthweight, even in the normal range (Shenkin et al., 2004) and longer duration of breastfeeding (Horta et al., 2015) correlate positively with cognitive outcomes. Parental psychopathology can influence parent‐child dynamics, ultimately contributing to individual differences in cognitive function (Valcan et al., 2018). Indeed, close parent‐child relationships in low‐SES students is associated with academic resilience (Kong, 2020). Finally, numerous aspects of the social and physical environment can influence children's cognitive outcomes. For instance, adverse childhood experiences, exposure to trauma, access to greenspaces, neighborhood walkability, traffic, and aspects of the school environment have all been shown to be associated with cognitive development (Lund et al., 2020; Piccolo et al., 2019; Sylvers et al., 2022; Vella‐Brodrick & Gilowska, 2022). However, few studies have examined whether these factors moderate associations between SES and cognitive function (Rakesh et al., 2024) and most work on these associations examines risk and protective factors at a single level of influence without considering others.

Importantly however, based on the definition of resilience by Masten (2014), and recent developments in the field (Masten et al., 2021), there is a need to understand the interplay between risk and protective factors at multiple levels in promoting resilience. However, most studies, including those in large samples (Yan & Gai, 2022), have examined only a few individual factors, typically separately. Consequently, the unique contribution of these different individual, home, and community‐level variables remains unknown. In addition, risk and protective factors may interact with one another. For example, given their roles in positive child outcomes (Goetschius et al., 2023; O’Malley et al., 2015; Raniti et al., 2022; Vella‐Brodrick & Gilowska, 2022), it is possible that positive school environments or access to greenspaces may buffer the risk conferred by household‐level factors. However, since studies examine individual factors, and typically in separate models, such interactions remain uninvestigated. Further, the protective role of other factors that may promote positive cognitive outcomes among children from low‐income families (e.g., neighborhood walkability) have yet to be investigated. As such, given that children live in complex socio‐ecological environments (Bronfenbrenner, 1994), there is a need for work that explores the independent and joint effects of a range of different individual, home, and community and school level factors. Such exploratory research is essential for understanding how children's behavior, temperament, family history, and environment contribute to cognitive resilience and for identifying modifiable targets for future interventions. Given the large number of children affected by economic hardship (ALICE in Focus, 2022; Annie E. Casey Foundation, 2014; Crouch et al., 2019), such investigations have critical public health relevance.

The aim of this exploratory study was to identify factors that promote cognitive resilience among youth from low‐income backgrounds in a large population‐based sample. To this end, we leveraged the Adolescent Brain Cognitive Development (ABCD) Study, a large population‐based cohort of children aged 9–10 years, and machine learning models to comprehensively investigate the role of child characteristics (including behaviors and activities, personality and temperament, physical and mental health), family history (such as parent mental health and developmental history), and environment (including the home environment, neighborhood environment, and traumatic experiences) in cognitive resilience among low‐income children.

## METHODS AND MATERIALS

### Participants

Study participants were from the ABCD study (baseline assessment; release 5.0). The ABCD study has enrolled over 11,500 children aged 9–10 years to comprehensively examine psychological and neurobiological development in a large sample (Garavan et al., 2018). The study's 21 sites were strategically chosen for demographic similarity to the overall US population. Within each site, participants were randomly selected from public, public charter, and private schools within a 50‐mile radius. Written informed consent was obtained from all caregivers, and all children provided assent. The rights of participants were protected under the local institutional review boards. The final sample included 3373 youth that met our inclusion criteria (see subsequent sections). These participants were 28.5% non‐Hispanic White, 28.6% non‐Hispanic Black, 30.3% Hispanic, 0.9% Asian, and 11.6% other race/ethnicity (which includes multiracial identities).

### Income‐to‐needs and cognitive performance

*Income‐to‐needs.* The income‐to‐needs ratio was calculated by dividing the median value of the income band by the federal poverty line corresponding to the household size. A value of 1 would indicate being exactly at the poverty threshold and values above and below 1 would indicate being above and below the threshold, respectively. Given our focus on cognitive resilience in the context of low SES, only participants in the bottom tertile (33%) of the income‐to‐needs distribution were included in analyses.

*Cognitive performance.* Cognitive performance was assessed using the NIH Toolbox Cognition Battery (Luciana et al., 2018). Age‐corrected composite scores of crystallized and fluid cognition were used in our analyses (Heaton et al., 2014; Luciana et al., 2018). Fluid and crystallized cognition were analyzed in separate models as they are distinct constructs (Cattell, 1963, 1987). Fluid cognition pertains to the capacities essential for abstract reasoning, while crystallized intelligence encompasses culturally acquired knowledge that is more likely to be environmentally determined (Cattell, 1963, 1987). The NIH Toolbox Cognition Battery assesses several cognitive abilities including attention, executive function, working memory, episodic memory, language, and processing speed (Akshoomoff et al., 2013; Luciana et al., 2018). The tasks include the List Sorting Working Memory, Dimensional Change Card Sort, Flanker Inhibitory Control and Attention, Picture Sequence Memory, and Pattern Comparison Processing Speed for assessing fluid cognitive Functioning, and Picture Vocabulary and Oral Reading Recognition for assessing crystallized cognitive functioning. Importantly, performance on these tasks has shown to be predictive of academic performance (Distefano et al., 2023).

### Cognitive resilience

Cognitive resilience is not a well‐defined concept in the literature (Haft et al., 2016; Sattler & Gershoff, 2019). Higher cognitive function—less common among youth from low‐income households—is a strong predictor of academic achievement and long‐term success. Based on Luthar et al. (2000) and recent findings by Sattler and Gershoff (2019) showing that "high threshold resilience" is most predictive of positive long‐term outcomes, we define resilience as the presence of high cognitive function in children from low‐income households. To this end, income‐to‐needs was divided into tertiles (high, medium, low), and youth from the bottom tertile with above average performance on fluid cognition or crystallized cognition were categorized as resilient (*N* = 1158 for fluid and 888 for crystallized) and below average fluid cognition or crystallized cognition scores were categorized as below‐average‐scoring (BAS; *N* = 2215 for fluid and 2485 for crystallized). Individuals in the resilient group had similar crystallized cognitive (*M*_Resilient_ = 119.32, *M*_High income_ = 113.09, *M*_BAS_ = 89.84) and fluid cognitive (*M*_Resilient_ = 108.58, *M*_High income_ = 100.38, *M*_BAS_ = 80.77) scores to those in the high income tertile. See the Supplement for distributions of cognitive scores for the three groups (eFigure 2a and 2b).

A rigid poverty threshold was not employed to identify “poor” families as it substantially underestimates the number of children living in poverty (e.g., 16% according to the federal poverty line). Many households, despite being employed, cannot afford essential costs like housing, childcare, food, healthcare, and transportation (ALICE in Focus, 2022). Given this, and the fact that the relationship between SES and cognitive outcomes is not confined to a specific threshold but rather exists along a continuum (with stronger associations at the lower end of the income distribution) (McLoyd, 1998), we chose to use the lowest tertile of income‐to‐needs in our analyses. Participants in the lowest tertile were indeed from poor or near poor families, with a mean income‐to‐needs ratio of 1.01 (SD = 0.60, min = 0.03, max = 2.02) and most families (95.7%) were below 200% of the poverty line. Distributions of income‐to‐needs for those in the lowest tertile versus highest tertile have been provided in the Supplement (eFigure 1a). Nonetheless, to ensure that results did not depend on the chosen threshold, we considered a quartile income threshold in sensitivity analyses.

### Predictor variables

**FIGURE 1:**
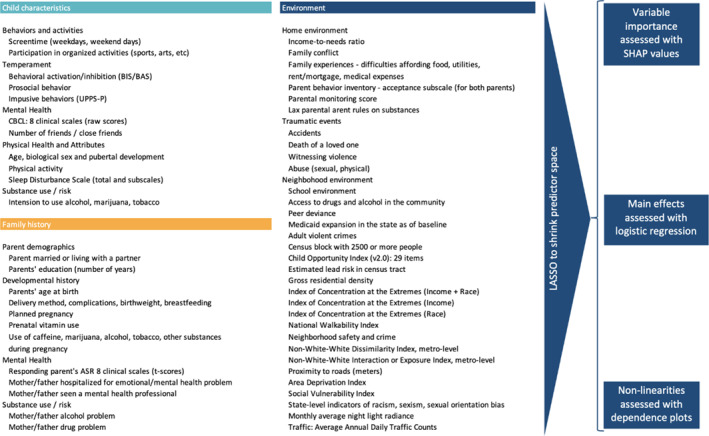
An overview of the predictor space and visual depiction of the methodological approach. The LASSO regression for each outcome (crystallized and fluid cognitive resilience) considers all listed potential predictors and shrinks the coefficients of variables that are irrelevant and redundant to zero. For each outcome, LASSO‐selected predictors are then included in XGBoost models, which allow for complex interactions and non‐linearities using a tree‐based prediction approach. Relative importance of predictor variables in the XGBoost models is assessed using SHAP values. Non‐linearities in the relationships between selected predictors and each outcome are assessed using dependence plots and estimates of linear relationships are summarized using odds ratios from logistic regressions.

A total of 164 predictor variables assessed child characteristics, family and developmental history, and environment (Figure 1; see Table S1 for a full list of predictors and their corresponding assessment methods). Child characteristics (41 variables) covered behaviors and activities (e.g., screentime, extra‐curricular activities), temperament (e.g., impulsive and prosocial behavior), and health (e.g., internalizing and externalizing symptoms, pubertal development, and sleep quality). Family and developmental history (32 variables) included parent demographics, maternal age at birth, birthweight, breastfeeding duration, and caregiver mental health. Finally, 91 variables assessed the child's environment, including the home environment (e.g., family conflict, financial adversity, caregiver warmth), history of traumatic events, and the neighborhood environment (e.g., school environment, childhood opportunity indices, crime rate, residential segregation, peer behaviors). The *mice* package in R (Buuren & Groothuis‐Oudshoorn, 2011) was used to impute missing values for the predictor variables through chained equations with predictive mean matching. This procedure imputes missing data through an iterative series of prediction models using other variables in the data set. It is frequently used in studies that employ machine learning analysis methods and demonstrates consistently low root mean squared error, even in longitudinal settings (Shaw et al., 2023).

### Prediction models

First, the data was split into 10 folds (with children from the same family retained in the same fold). We ran nested 10‐fold cross‐validated machine learning classification analyses to predict crystallized and fluid cognitive resilience outcomes (in separate models) using the predictor variables, which were all included in the model simultaneously. The first analytical step involved applying LASSO (Least Absolute Shrinkage and Selection Operator) regression. LASSO is a regularization technique that helps to mitigate multicollinearity and prevent overfitting by shrinking some regression coefficients to zero, effectively excluding less relevant predictors from the model. This process results in a more parsimonious model with only the most influential variables retained. However, while LASSO aids in predictor selection and reduces redundancy, estimates obtained from LASSO are biased as it applies regularization. To address this, we next utilized logistic regression with the predictors selected by LASSO (i.e., those with non‐zero coefficients). Logistic regression is used for binary outcome variables and provides estimates of the odds ratios, which reflect the strength and direction of the relationship between each predictor and the outcome.

Lastly, Extreme Gradient Boosting (XGBoost; R package xgboost; Chen & Guestrin, 2016) classification was used to build a prediction model with the LASSO‐selected predictors to incorporate potential interactions and non‐linearities in associations and ascertain relative variable importance (Lundberg & Lee, 2017). XGBoost builds an ensemble of decision trees sequentially, where each tree corrects the errors of its predecessors. This approach captures complex interactions and non‐linear relationships between predictors and outcomes. Variable importance was assessed using SHapley Additive explanation (SHAP) values. SHAP values provide an understanding of each predictor's contribution to the model's predictions. SHAP values ensure that each predictor's impact is assessed by considering all possible combinations of predictors. Each SHAP value represents how much a specific predictor changes the model's prediction compared to the average prediction if that predictor were not included. By aggregating these individual contributions, SHAP values provide a measure of each variable's overall importance. This means that the contribution of a predictor is evaluated not in isolation, but in the context of other predictors. Specifically, positive and negative SHAP values indicate that the feature increases and decreases the predicted value, respectively. SHAP values inherently capture non‐linearity and can thus be used to examine the dependence of the prediction on each predictor across the range of its values in dependence plots. See Figure 1 for a visual depiction of the approach.

The above approach was selected from a number of strategies including the direct application of XGBoost and SuperLearner ensemble classification to the full set of predictors. Since accuracy did not differ substantially between strategies (see Table S2), results from LASSO regression followed by XGBoost classification are reported in the main manuscript due to maximal interpretability and accuracy (as assessed through area under‐the‐curve [AUC]).

### Sensitivity analyses

In addition to testing a more conservative threshold to define the low‐income group (namely the lowest quartile rather than lowest tertile of the income‐to‐needs distribution), we also repeated the main analysis to discriminate between those with above average cognitive function and those with below average cognitive function among youth from high income backgrounds (i.e., highest income tertile). This analysis allowed us to determine which factors play a role in promoting cognitive function specifically in a low‐income context, as opposed to promoting high cognitive function generally.

Lastly, we ran a sensitivity analysis accounting for clustering within families. The code for all analyses conducted is shared in the following repository: https://github.com/katsadikova/ABCD_cog_resilience.git.

## RESULTS

### Demographic information

The sample used in the main analysis comprised 3373 children with a mean age of 9.47 ± 0.51 years. See Table 1 for demographic information on resilient and BAS children.

**TABLE 1:** Sample descriptives.

	Fluid cognition		Crystallized cognition	
	Resilient	BAS	Resilient	BAS
*n* (%)	1158 (34.3%)	2215 (65.7%)	888 (26.3%)	2485 (73.7%)
Age, years	9.47 ± 0.51	9.45 ± 0.51	9.49 ± 0.51	9.45 ± 0.51
Female sex	48%	49%	44%	50%
Income‐to‐needs	1.12 ± 0.58	0.95 ± 0.59	1.21 ± 0.56	0.94 ± 0.59
Fluid cognitive functioning	105.09 ± 17.39	93.69 ± 14.67	119.32 ± 12.48	89.84 ± 9.38
Crystallized cognitive functioning	108.59 ± 10.43	80.78 ± 10.50	98.82 ± 17.05	87.29 ± 15.71
Total cognitive functioning	107.96 ± 13.42	84.38 ± 12.22	110.58 ± 14.19	86.00 ± 12.50

*Note*: Values indicate mean ± standard deviation unless otherwise specified. BAS = below‐average‐scoring.

### Model performance

The LASSO regression model had good discrimination between resilient and BAS children but was substantially more accurate for crystallized cognition (AUC = 0.75) than for fluid cognition (AUC = 0.67).

### Predictors of cognitive resilience

**FIGURE 2:**
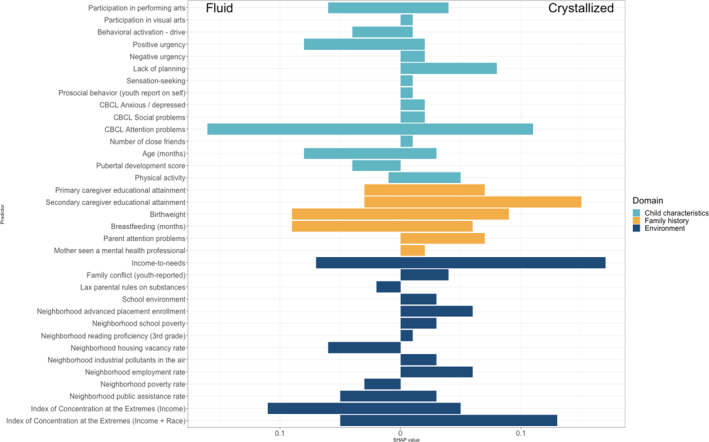
Variable importance for predicting resilience for fluid (left) and crystallized cognitive (right) across three domains. The three colors represent the three domains of the predictors: teal for child characteristics, yellow for family and developmental history, and blue for environment. This figure displays the absolute SHAP value for each predictor to indicate variable importance. SHAP values provide an understanding of each predictor's contribution to the model's predictions. Each SHAP value represents how much a specific predictor changes the model's prediction compared to the average prediction if that predictor were not included. Positive and negative SHAP values indicate that the feature increases and decreases the predicted value, respectively. For each feature, average absolute SHAP values across individuals reflect the marginal contribution of the feature across all possible feature combinations, thus serving as a quantitative measure of its importance.

Overall, 35 of the 164 considered factors across child characteristics, family and developmental history, and environment contributed meaningfully to the prediction of both fluid and crystallized cognitive resilience (see Figure 2 for SHAP values representing relative predictor importance and Table 2 for odds ratios for LASSO‐selected predictors).

**TABLE 2:** Odds ratios for predictors of fluid and crystallized cognitive resilience among children from households within the lowest INR tertile (*N* = 3373).

		Fluid cognitive resilience			Crystallized cognitive resilience		
Child characteristics		OR	95% CI	*P*	OR	95% CI	*P*
Behavior and activities	Participation in performing arts^1^	1.28*	(1.09,1.50)	<0.001	1.28*	(1.07,1.53)	0.007
	Participation in visual arts^2^				1.32*	(1.06,1.64)	0.01
Temperament	Behavioral activation ‐ drive	0.97*	(0.95,1.00)	0.028	0.98	(0.96,1.01)	0.28
	Positive urgency	0.96*	(0.94,0.99)	<0.001	0.97*	(0.94,1.00)	0.04
	Negative urgency				0.96*	(0.93,1.00)	0.04
	Lack of planning				1.04*	(1.00,1.08)	0.03
	Sensation‐seeking				1.06*	(1.03,1.10)	<0.001
	Prosocial behavior (youth report on self)^2^				0.72*	(0.56,0.91)	0.006
Mental health	CBCL: Anxious/depressed				1.08*	(1.04,1.11)	<0.001
	CBCL: Social problems^2^				0.92*	(0.87,0.97)	0.002
	CBCL: Attention problems	0.93*	(0.91,0.95)	<0.001	0.91*	(0.88,0.94)	<0.001
	Number of close friends				0.99*	(0.98,1.00)	0.03
Physical health and attributes	Age (months)	1.02*	(1.01,1.03)	<0.001	1.01*	(1.00,1.02)	0.03
	Pubertal development score^1^	0.89*	(0.81,0.97)	0.006			
	Physical activity^1^ ^,^ ^2^	1.04*	(1.01,1.08)	0.01	1.06*	(1.02,1.10)	0.002
Family history							
Demographics	Primary caregiver educational attainment	1.05*	(1.00,1.1)	0.03	1.04	(0.99,1.10)	0.12
	Secondary caregiver educational attainment	1.02	(0.98,1.06)	0.35	1.08*	(1.03,1.13)	<0.001
Developmental history	Birthweight	1.11*	(1.06,1.18)	<0.001	1.15*	(1.09,1.23)	<0.001
	Breastfeeding (months)	1.01	(1.00,1.02)	0.08	1.01*	(1.00,1.02)	0.04
	Parent attention problems^2^				1.04*	(1.02,1.05)	<0.001
	Mother seen a mental health professional				1.21	(1.00,1.47)	0.05
Environment							
Home environment	Income‐to‐needs^1^	1.16*	(1.01,1.33)	0.04	1.5*	(1.28,1.76)	<0.001
	Family conflict (youth‐reported)^2^				0.94*	(0.90,0.98)	0.007
	Lax parental rules on substances^1^	0.91*	(0.86,0.97)	<0.001			
Neighborhood	School environment				0.96*	(0.94,0.99)	0.02
	Neighborhood advanced placement enrollment^1^ ^,^ ^2^				1.94*	(1.32,2.87)	<0.001
	Neighborhood school poverty^2^				1.00	(0.99,1.00)	0.83
	Neighborhood reading proficiency (3rd grade)				1.00	(1.00,1.00)	0.40
	Neighborhood housing vacancy rate^1^	0.98*	(0.97,1.00)	0.02			
	Neighborhood industrial air pollutants^2^				1.06*	(1.03,1.09)	<0.001
	Neighborhood employment rate^2^				1.01	(1.00,1.02)	0.08
	Neighborhood poverty rate^1^	0.99	(0.98,1.00)	0.21			
	Neighborhood public assistance rate^1^ ^,^ ^2^	1.00	(0.99,1.01)	0.42	1.00	(0.99,1.01)	0.71
	Index of Concentration at the Extremes (Income)^2^	1.01	(0.53,1.93)	0.97	1.18	(0.60,2.32)	0.64
	Index of Concentration at the Extremes (Income + Race)^1^ ^,^ ^2^	1.14	(0.55,2.36)	0.72	1.33	(0.56,3.17)	0.52

Abbreviation: CBCL, Child Behavior Checklist.

^*^ Two‐sided *p*‐value < 0.05.

Based on results from the sensitivity analysis:

^1^ Predictors for fluid cognitive resilience in the low‐income, but not in the high‐income analysis.

^2^ Predictors for crystallized cognitive resilience in the low‐income, but not in the high‐income analysis.

#### Common predictors for fluid and crystallized cognitive resilience

**FIGURE 3:**
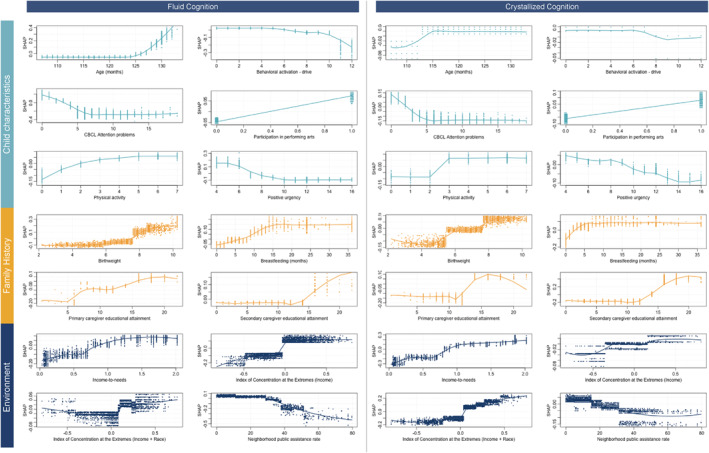
Dependence plots for predictors common to fluid (left) and crystallized cognitive resilience (right). A dependence plot visualizes the relationship between a single predictor's value (shown on the *x*‐axis of each panel) and its SHAP value (shown on the *y*‐axis), which represents the impact of that predictor on the model's prediction. In these plots, a higher SHAP value indicates that the predictor is positively contributing to cognitive resilience, while a lower SHAP value suggests a negative contribution. For example, if a plot shows that as the predictor value increases, the SHAP value also increases, this suggests that higher values of that predictor are associated with greater cognitive resilience. The three colors represent the three domains of the predictors: teal for child characteristics, yellow for family and developmental history, and blue for environment.

Of the 35 LASSO‐selected predictors, 14 were common between crystallized and fluid cognitive outcomes and included six child characteristics, four family history markers, and four environmental factors (Figure 3). Among child characteristics, higher frequency of physical activity and participation in performing arts activities were associated with greater odds of resilience. Lower levels of positive urgency and lower attention symptoms were found to increase the odds of resilience. Finally, older children were also more likely to be classified as resilient.

As for family history, longer duration of breastfeeding and higher birthweight were associated with higher odds of resilience. However, the benefits with respect to crystallized cognition plateaued after 5 months of breastfeeding, while the potential benefits to fluid cognition persisted to approximately 15 months. Further, while primary and secondary caregiver educational attainment were both relevant for resilience, the former was more important for fluid cognition and the latter for crystallized cognition.

At the neighborhood level, lower public assistance rates and living in higher privilege also predicted resilience. Finally, higher income even within the bottom tertile had benefits for resilience, particularly with respect to crystallized cognition.

#### Unique predictors of fluid cognitive resilience

**FIGURE 4:**
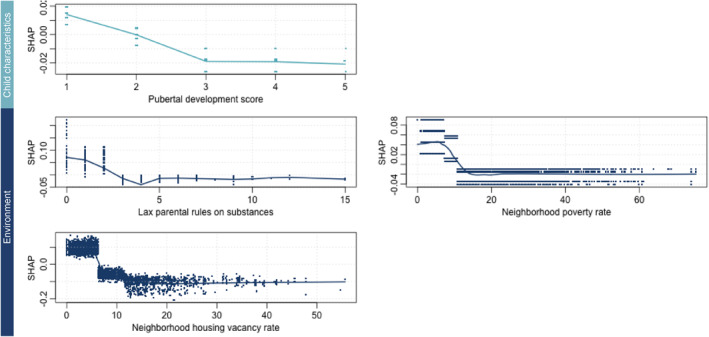
Dependence plots for predictors unique to fluid cognition resilience. A dependence plot visualizes the relationship between a single predictor's value (shown on the *x*‐axis of each panel) and its SHAP value (shown on the *y*‐axis), which represents the impact of that predictor on the model's prediction. In these plots, a higher SHAP value indicates that the predictor is positively contributing to cognitive resilience, while a lower SHAP value suggests a negative contribution. For example, if a plot shows that as the predictor value increases, the SHAP value also increases, this suggests that higher values of that predictor are associated with greater cognition resilience. The colors represent the domains of the predictors: teal for child characteristics and blue for environment.

Resilience in the context of fluid cognition was uniquely predicted by less advanced pubertal development, stricter parental rules around substance use in the household, and lower vacancy and poverty rates in the neighborhood (Figure 4).

#### Unique predictors of crystallized cognitive resilience

**FIGURE 5:**
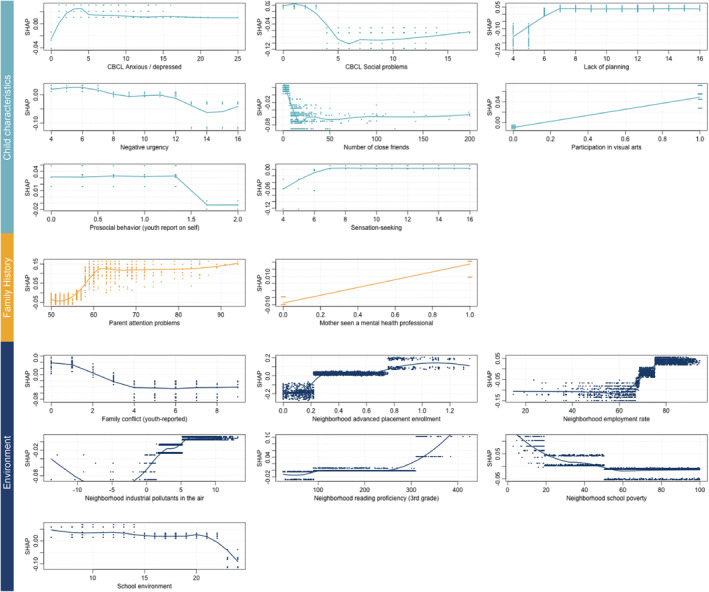
Dependence plots for predictors unique to crystallized cognitive resilience. A dependence plot visualizes the relationship between a single predictor's value (shown on the *x*‐axis of each panel) and its SHAP value (shown on the *y*‐axis), which represents the impact of that predictor on the model's prediction. In these plots, a higher SHAP value indicates that the predictor is positively contributing to cognitive resilience, while a lower SHAP value suggests a negative contribution. For example, if a plot shows that as the predictor value increases, the SHAP value also increases, this suggests that higher values of that predictor are associated with greater cognitive resilience. The three colors represent the three domains of the predictors: teal for child characteristics, yellow for family and developmental history, and blue for environment.

A much larger number of factors predicted resilience uniquely in the context of crystallized cognition (Figure 5). Among child characteristics and behavior, participation in visual arts improved the odds of resilience. Other relationships were slightly more complex. Lower negative urgency, some sensation seeking and some degree of spontaneity – or lack of planning – promoted resilience. However, higher self‐ratings of prosocial behaviors, reporting a large number of close friendships (more than 20), experiencing higher levels of social problems, and the complete absence of parent‐reported youth anxiety or depression symptoms were associated with lower odds of resilience.

In terms of family history, children with a parent reporting higher levels of attention symptoms and seeking assistance from a mental health professional were associated with crystallized cognitive resilience. Lastly, for environment, lower family conflict, living in neighborhoods with higher enrollment in advanced placement courses, higher neighborhood employment rates, stronger third grade reading proficiency, and lower school poverty promoted greater odds of crystallized cognitive resilience. However, self‐reported highly positive school environments were associated with lower odds of resilience.

### Sensitivity analyses

We found the results to be stable when the low‐income group was defined as those in the bottom quartile (rather than tertile) of the income‐to‐needs distribution. Of the top 10 predictors identified in the main analysis, 9 were also selected as important predictors in the sensitivity analysis for both fluid and crystallized cognition (see the Tables S5a and S5b in the Supplement for the list of predictors).

Comparisons of predictors of above‐average crystallized and fluid cognition in the low‐income and high‐income samples are summarized in the Supplement in Tables S4a and S4b, respectively. Our findings revealed that certain factors, such as caregiver education levels and the duration of breastfeeding, were associated with high cognitive performance in both income groups. However, several factors were unique to the low‐income group (marked with footnotes in Table 2). Among the factors that were common to both low‐income and high‐income analyses, they were less prevalent or, in the case of negative factors, more prevalent in the low‐income group (e.g., duration of breastfeeding is longer in high‐income families), evidenced by the mean and SD values reported in Table S4a and S4b.

Correcting the main analysis for clustering within families demonstrated little impact on the estimated odds ratios and their standard errors (compare results reported in Table 2 to those reported in Table S3 in the Supplement).

## DISCUSSION

The goal of this study was to comprehensively characterize predictors of cognitive resilience during late childhood. Using aspects of youth behavior and activities, family and developmental history, and the environment, we were able to successfully discriminate between cognitively resilient and BAS children. The predictive accuracy and discrimination achieved by our primary modeling strategy is comparable to other work employing machine learning strategies for classification problems in social and behavioral science (Papini et al., 2023). Prediction accuracy was higher for crystallized than fluid cognitive resilience and a larger number of predictors were found to predict crystallized cognitive resilience.

To identify factors that specifically enhance cognitive function in low‐income contexts, rather than those that generally support high cognitive performance, we conducted additional analyses focusing on high‐income youth (i.e., the highest income tertile) to differentiate between individuals with above‐average and below‐average cognitive function. These analyses revealed that while some factors, such as primary and secondary caregiver education and longer breastfeeding duration, contribute to high cognitive performance across both income groups, several factors were uniquely predictive of cognitive resilience in the low‐income group. These factors included participation in visual and performing arts, pubertal development, physical activity, income‐to‐needs ratio, family conflict, and various neighborhood‐level factors, such as poverty rate, school poverty, and advanced placement enrollment. These factors could be considered as resilience‐promoting factors, as they seem to specifically enhance cognitive function in low‐income settings. However, even among the factors that were common across income levels, we observed disparities in their prevalence between low‐ and high‐income groups. For instance, positive factors like longer breastfeeding duration were less common among low‐income youth, while negative factors, such as mental health problems, were more prevalent. This underscores the importance of promoting these factors in low‐income contexts, even though they are beneficial across income levels, and are therefore discussed in this paper.

Several factors predicted both fluid and crystallized cognitive resilience. Notably, even though all children were in the lowest tertile of income‐to‐needs, small incremental increases in income‐to‐needs had a discernible association with resilience. This is consistent with studies showing that changes in income have particularly meaningful effects on children's cognitive and schooling outcomes at the lower end of the income spectrum (Cooper & Stewart, 2013). Similarly, consistent with prior work (Davis‐Kean, 2005), higher caregiver educational attainment was associated with higher odds of resilience. Other factors included children's temperament, attention problems, extracurricular activities, and developmental history—including breastfeeding, and birthweight. Importantly, more frequent moderate to intense physical activity and participation in performing arts increased the odds of cognitive resilience. Our findings are consistent with intervention studies showing the benefits of physical activity on cognitive outcomes (Haverkamp et al., 2020) and highlight the importance of physical activity, particularly among children from low‐income backgrounds (Foster & Marcus Jenkins, 2017). Even after accounting for a wide range of other factors, participation in visual and performing arts was associated with resilience, which stands in contrast to prior null findings (Foster & Marcus Jenkins, 2017). Future intervention studies should investigate whether participation in extracurricular activities supports cognitive function among children from low‐income families.

Children with higher attention problems and lower birthweight were less likely to be classified as resilient. Given that attention problems are more prevalent in low‐SES youth (Russell et al., 2016), there is a need for targeted interventions specifically designed for children grappling with attention issues. Moreover, future work should aim to pinpoint effective strategies to promote positive cognitive outcomes among children born with low birthweight. Finally, longer duration of breastfeeding was also associated with higher odds of cognitive resilience. In line with advice by the World Health Organization, our findings highlight the importance of providing support to women from low‐income households post birth (e.g., paid maternity leave, dedicated spaces for breastfeeding in the workplace and public places, flexible working days) and increasing public messaging around the importance of breastfeeding to improve cognitive outcomes among children from low‐income households.

Fluid cognitive resilience was uniquely predicted by a few factors including pubertal development, parental rules on substance use, and neighborhood‐level factors. Specifically, less advanced pubertal status was associated with higher odds of fluid cognitive resilience. This finding aligns with the hypothesis that exposure to chronic stress, which is more common in low‐income contexts, can trigger faster pubertal development (Ellis et al., 2022), contributing to lower cognitive performance (Stumper et al., 2020). Further, stricter parental rules on substance use are positively associated with fluid cognitive resilience. Rather than being a mechanism of resilience, higher parental monitoring regarding substance use may be a proxy for other factors (e.g., parental expectations) that support cognitive resilience (Yan & Gai, 2022) but were not measured in the present study.

Factors across all three domains uniquely predicted crystallized cognitive resilience. For example, lower levels of social problems and having a small circle of close friends was associated with higher odds of resilience. Previous research has shown that peer support promotes resilient functioning in psychosocial domains (Harmelen et al., 2017), which may in turn contribute to the positive cognitive outcomes observed in this study. This aligns with work showing links between school connectedness and belonging and wellbeing (Raniti et al., 2022). Future work should directly test whether promoting greater integration in classrooms and facilitating friendships through play and activities during and after school benefits the cognitive outcomes of children from low‐income households. Additionally, lower levels of family conflict were associated with greater odds of crystallized cognitive resilience. Consistent with prior work that indicates that stress could influence the development of physiological systems that regulate attention (Hinnant et al., 2013), our results suggest that a family environment characterized by conflict may hinder a child's ability to focus, learn, and retain information.

Various neighborhood‐level factors emerged as predictors of fluid and crystallized cognitive resilience. Specifically, living in neighborhoods characterized by higher privilege was associated with higher odds of resilience across both domains. Further, lower levels of neighborhood and school poverty, housing vacancy rates, and neighborhood‐level academic proficiency—including advanced placement enrollment and third‐grade proficiency—were associated with higher odds of crystallized cognitive resilience. These findings underscore the importance of neighborhood environments for cognitive development (Lloyd et al., 2010), which likely influence child development through multiple pathways. For example, in addition to structural community characteristics such as quality of education, advantaged neighborhoods are also associated with parenting behaviors such as the utilization of education‐focused practices (Burton & Robin, 2000; Greenman et al., 2011; Klebanov et al., 1994; Shumow & Lomax, 2009), which may contribute to cognitive resilience among low‐income children. Further, the presence of high‐achieving peers who excel in reading and enroll in advanced placement classes can model behaviors that foster crystallized cognitive resilience. Future efforts could explore how enhancing access to early childhood education programs and advanced placement courses, particularly in resource‐deprived neighborhoods, might support the development cognitive resilience among children from low‐income backgrounds.

Some findings were more challenging to interpret. For example, fluid cognitive resilience was associated with not having very high levels of behavioral drive, positive urgency, and planning. Further, not having very low levels of sensation seeking was also associated higher odds of resilience. These findings demonstrate that children from low‐income households who are open to some degree of novelty and stimulation, less impulsive in response to positive emotions, and exhibit moderate or balanced level of motivation, may demonstrate better cognitive resilience. Further, the dependence plots indicate that these associations are likely driven by non‐linearities at the tails of the predictor distributions but are close to null when the values of these predictors that are most common in the sample are considered. Similarly, our results show that having a primary caregiver with higher self‐reported attention symptoms and a history of mental health professional consultations increases odds for crystallized cognitive resilience. These factors likely do not function as direct mechanisms of resilience. Instead, it is possible that children in households where the primary caregiver acknowledges and addresses their own mental health challenges—manifested through attention problems—and is proactive in seeking professional support tend to fare better cognitively.

Some limitations should be considered when interpreting findings. First, the study was cross‐sectional. Therefore, we cannot comment on directionality or causality and any clinical implications are purely speculative. Longitudinal and intervention studies with rigorous adjustment for measured confounders and assessment of the influence of unmeasured confounding within a causal framework are needed. Of note, the present study leveraged baseline data as it was not possible to use composite scores from post‐baseline visits since the entire cognitive battery was not administered at subsequent time‐points (see ABCD release notes). Second, it is unknown whether resilience is transient or persistent. Future longitudinal work should assess factors that predict cognitive resilience across time‐points. Third, given the major role of neighborhood level factors, it is unknown whether these findings generalize. Similar investigations will need to be conducted using data from other populations. Four, the odds ratios from the logistic regression models need to be interpreted with caution in instances where non‐linearities are evident in dependence plots. Such odds ratios are nonetheless useful for approximating effect sizes. Five, some of the identified associations were challenging to interpret – for example, evidence that odds of resilience are higher in the most supportive school environments or in neighborhoods with higher airborne pollutant levels. These findings may be due to measurement error or skew in predictor distributions (e.g., most children living in urban polluted environments). Future research should aim to characterize these associations more thoroughly. Six, several studies, including ours, have examined associations between SES and brain structure and function (Rakesh et al., 2022, 2023; Rakesh & Whittle, 2021; Rakesh, Zalesky, & Whittle, 2021). Evidence suggests that positive environmental factors can mitigate the impact of low SES on brain outcomes (Brody et al., 2019; Rakesh, Seguin, et al., 2021; Whittle et al., 2017), and distinct neurobiological profiles have also been identified in youth from low‐income backgrounds who show positive outcomes (Ellwood‐Lowe et al., 2022). However, few studies have investigated the neurobiology underlying cognitive resilience among low‐income youth. Future work should use a machine learning approach to identify brain features that may predict such resilience. Given that socioeconomic gaps in achievement have not declined over several decades despite substantial efforts, this research is crucial for identifying novel targets for intervention. Seven, while efforts were made to make the ABCD sample representative, parents in this sample have higher income‐to‐needs and educational attainment. As such, the bottom tertile of the ABCD sample may not reflect the bottom tertile of the national population, thus impacting the generalizability of the results. Eight, although the NIH toolbox includes seven tasks that measure a range of cognitive abilities, including attention, executive function/cognitive flexibility, working memory, episodic memory, language, and processing speed, and are associated with academic performance (Distefano et al., 2023), factors that promote resilience in other cognitive domains (e.g., sustained attention) may have been missed. Nine, our study focused on a specific set of predictors. There are several other factors that may promote cognitive resilience such as access to mental health services, nutrition, social support networks, and special needs education that were not assessed in the present sample. Future work should consider these and other factors we did not assess as predictors of cognitive resilience. Ten, the machine learning methods used in our study, while powerful in identifying patterns and relationships within the data, are inherently atheoretical. Future research could benefit from an approach that integrates machine learning with theory‐driven hypotheses. This would involve using machine learning to identify potential relationships and then applying theoretical frameworks to interpret and validate these findings. Such an approach could provide a more nuanced understanding of cognitive resilience and its underlying mechanisms. Finally, while some of the effect sizes in this study were small, small effects can accumulate over time and are meaningful at the population level (Funder & Ozer, 2019).

This study provides a comprehensive characterization of the predictors of cognitive resilience during adolescence. Using a large, diverse sample of children from low‐income households, we found that several factors across all three domains of individual, family, and neighborhood characteristics predict both fluid and crystallized cognitive resilience. Our findings highlight the importance of a multifaceted approach to promoting cognitive resilience among children from low‐income households. Future intervention work should examine the role of modifiable factors such as physical activity opportunities, access to and encouragement of involvement in extracurricular activities, support for women during pregnancy and postpartum, and high‐quality early childhood education. These efforts can help to improve the long‐term outcomes for children from low‐income households.

## AUTHOR CONTRIBUTION

Divyangana Rakesh: Conceptualization (lead); Funding acquisition (lead); Methodology (equal); Project administration (lead); Writing – Original Draft (lead); Writing – Review & Editing (equal). Ekaterina Sadikova: Methodology (equal); Formal analysis (lead); Writing – Original Draft (supporting); Writing – Review & Editing (equal). Katie A. McLaughlin: Supervision (lead); Writing – Review & Editing (equal).

## CONFLICT OF INTEREST STATEMENT

The authors report no biomedical financial interests or potential conflicts of interest.

## ETHICAL CONSIDERATIONS

The need for ethics approval was waived by The University of California, Los Angeles, institutional review board (IRB) stating that secondary analyses using the publicly released ABCD Study data are not human subjects research and therefore do not require their own approval. The ABCD Study received their own central IRB approval. All guidelines pertaining to the Declaration of Helsinki were adhered to. Caregivers provided written informed consent and children provided assent for participation in the study.

## Supporting information

Supplementary Material

## Data Availability

The Adolescent Brain Cognitive Development (ABCD) Study data is available for qualified researchers who have an approved Data Use Certificate (DUC) from the National Institute of Mental Health (NIMH) Data Archive (NDA).
