# Supplementary material for: Beyond the income‐achievement gap: The role of individual, family, and environmental factors in cognitive resilience among low‐income youth
Source: JCPP Adv. 2024 Dec 20;5(3):e12297. doi: 10.1002/jcv2.12297 (PMC12446723; doi:10.1002/jcv2.12297)
Supplement: Supplementary file 1 — Supplementary Material [file JCV2-5-e12297-s001.docx]

**Supporting Information***“Beyond the income-achievement gap: The role of individual, family, and environmental factors in cognitive resilience among low-income youth”*

Table of Contents

[Figure S1a. Distributions of income-to-needs within the low and high tertile of income-to-needs 2](#_Toc182212964)

[Figure S1b. Distributions of income-to-needs within the low and high quartile of income-to-needs 3](#_Toc182212965)

[Figure S2a. Comparison of average crystallized cognitive performance in the resilient and non-resilient groups in the lowest income-to-needs tertile and the average crystallized cognitive performance in the top income-to-needs tertile 4](#_Toc182212966)

[Figure S2b. Comparison of average fluid cognitive performance in the resilient and non-resilient groups in the lowest income-to-needs tertile and the average fluid cognitive performance in the top income-to-needs tertile 5](#_Toc182212967)

[Table S1. Detailed list of measures 6](#_Toc182212968)

[Table S2. Comparison of model discrimination using AUC by modeling strategy and outcome 14](#_Toc182212969)

[Table S3. Odds ratios for retained predictors from models accounting for familial clustering 15](#_Toc182212970)

[Table S4a. Comparison of predictors selected as important for prediction of crystallized resilience in the low income-to-needs tertile and high income-to-needs tertile samples 17](#_Toc182212971)

[Table S4b. Comparison of predictors selected as important for prediction of fluid resilience in the low income-to-needs tertile and high income-to-needs tertile samples 19](#_Toc182212972)

[Table S5a. Common predictors of crystallized cognitive performance in the bottom tertile and bottom quartile of the income-to-needs distribution 20](#_Toc182212973)

[Table S5b. Common predictors of fluid cognitive performance in the bottom tertile and bottom quartile of the income-to-needs distribution 21](#_Toc182212974)

### Figure S1a. Distributions of income-to-needs within the low and high tertile of income-to-needs


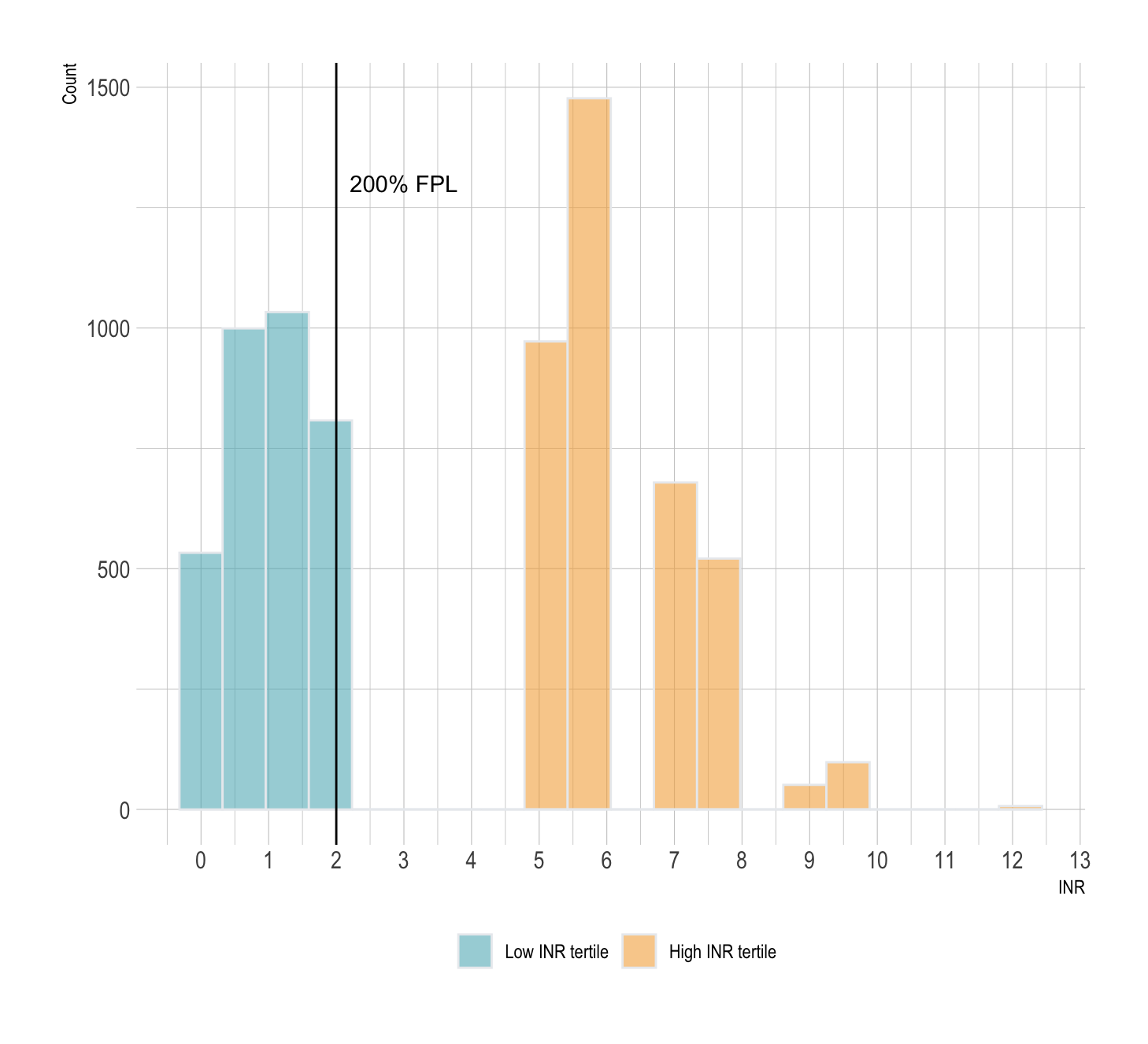


### Figure S1b. Distributions of income-to-needs within the low and high quartile of income-to-needs


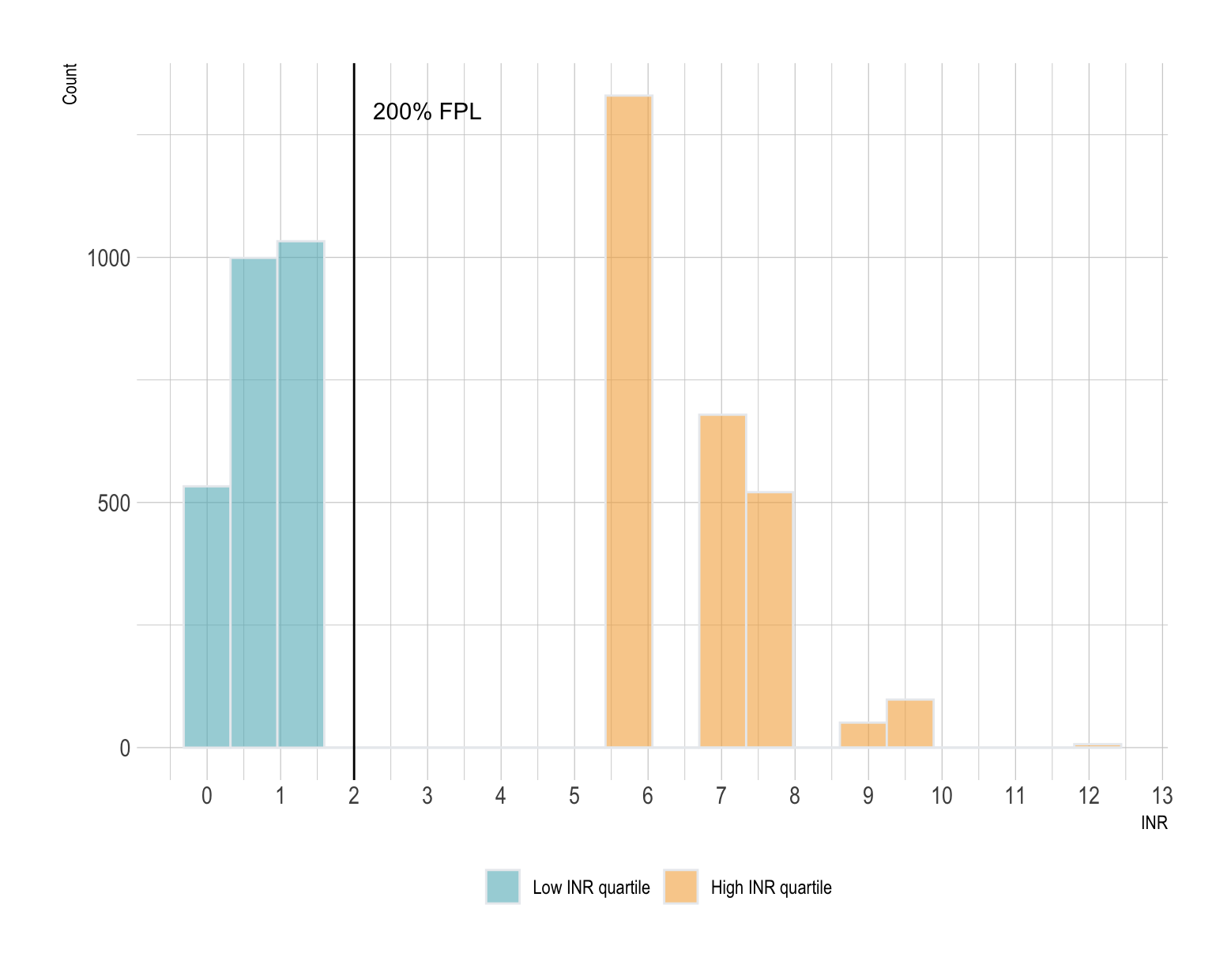


### Figure S2a. Comparison of average crystallized cognitive performance in the resilient and non-resilient groups in the lowest income-to-needs tertile and the average crystallized cognitive performance in the top income-to-needs tertile


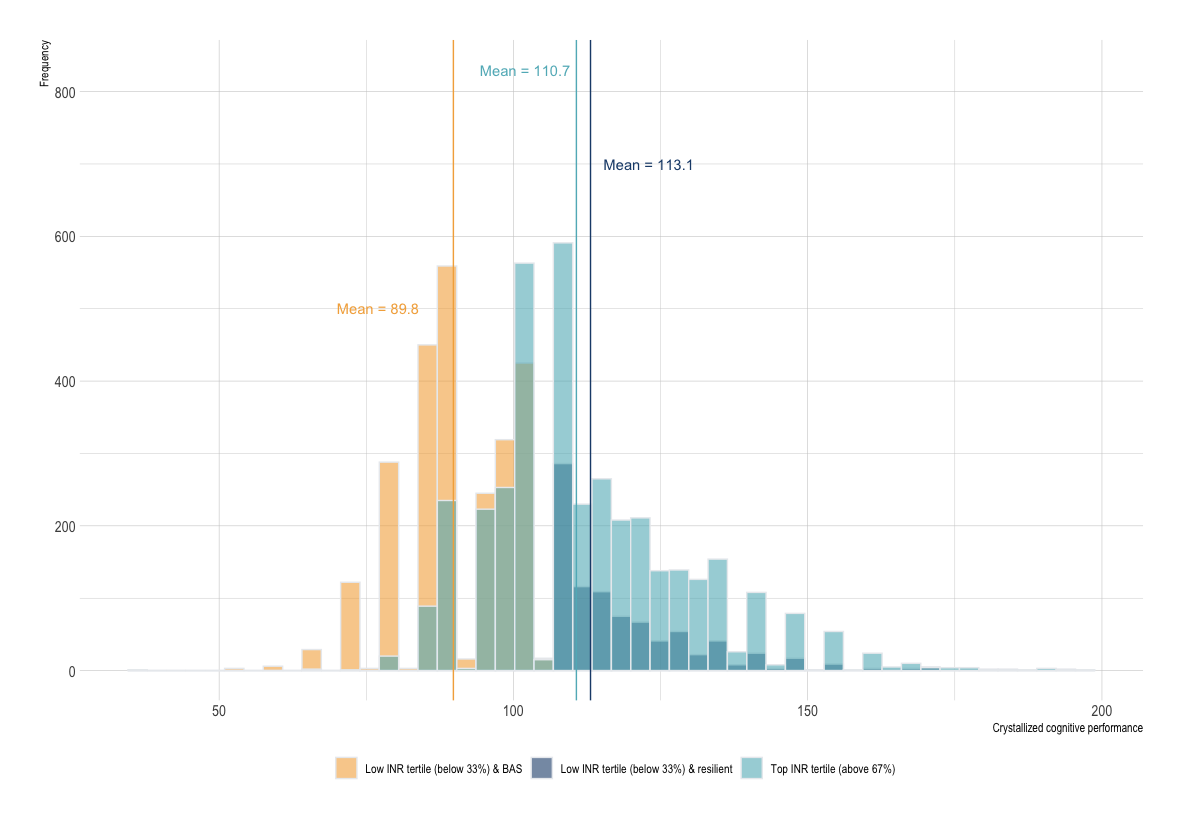


T-test comparing average crystallized cognitive performance scores in the top income-to-needs tertile versus the low income-to-needs tertile & resilient: t=-12.4, df=1,788.5, two-sided p-value<0.0001

T-test comparing average crystallized cognitive performance scores in the top income-to-needs tertile versus the low income-to-needs tertile & BAS: t=68.6, df=6,094.1, two-sided p-value<0.0001

### Figure S2b. Comparison of average fluid cognitive performance in the resilient and non-resilient groups in the lowest income-to-needs tertile and the average fluid cognitive performance in the top income-to-needs tertile


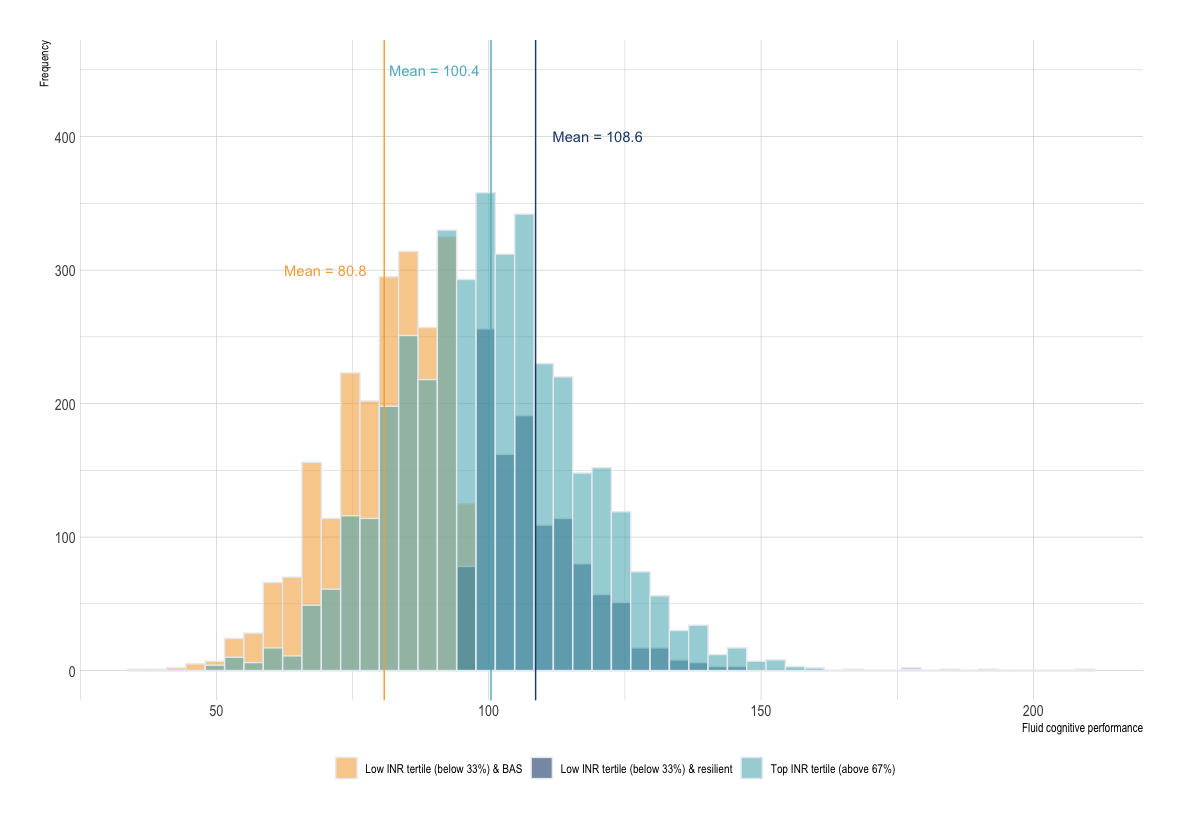


T-test comparing average fluid cognitive performance scores in the top income-to-needs (INR) tertile and in the low income-to-needs tertile & resilient: t=-20.0 df=3,125, two-sided p-value<0.0001

T-test comparing average fluid cognitive performance scores in the top income-to-needs tertile and in the low income-to-needs & BAS: t=55.6, df=5,991.3, two-sided p-value<0.0001

### Table S1. Detailed list of measures

| **Domain** | **Sub-domain** | **Variable** | **Scale/categories** | **Reporter** |
| --- | --- | --- | --- | --- |
| Child characteristics | Behavior and activities | Hours of screentime, typical weekday | Hours | Parent |
|  |  | Hours of screentime, typical weekend day | Hours | Parent |
|  |  | Participation in performing arts | 1=Yes, 0=No | Parent |
|  |  | Participation in visual arts | 1=Yes, 0=No | Parent |
|  |  | Participation in team sports | 1=Yes, 0=No | Parent |
|  |  | Participation in non-team sports | 1=Yes, 0=No | Parent |
|  |  | Participation in other activities | 1=Yes, 0=No | Parent |
|  |  | Participation in any organized activities | 1=Yes, 0=No | Parent |
|  | Temperament | BIS/BAS^1^: Behavioral activation - drive | Score ranging from 0 to 12 | Youth |
|  |  | BIS/BAS^1^: Behavioral activation - fun-seeking | Score ranging from 0 to 12 | Youth |
|  |  | BIS/BAS^1^: Behavioral activation - reward responsiveness | Score ranging from 0 to 15 | Youth |
|  |  | BIS/BAS^1^: Behavioral inhibition | Score ranging from 0 to 21 | Youth |
|  |  | UPPS-P^2^: Positive urgency | Score ranging from 4 to 16 | Youth |
|  |  | UPPS-P^2^: Negative urgency | Score ranging from 4 to 16 | Youth |
|  |  | UPPS-P^2^: Lack of premeditation | Score ranging from 4 to 16 | Youth |
|  |  | UPPS-P^2^: Lack of perseverance | Score ranging from 4 to 16 | Youth |
|  |  | UPPS-P^2^: Sensation-seeking | Score ranging from 4 to 16 | Youth |
|  |  | Prosocial behavior (parent report on youth)^3^ | Score ranging from 0 to 2 | Youth |
|  |  | Prosocial behavior (youth report on self)^4^ | Score ranging from 0 to 2 | Youth |
|  | Mental Health | Child anxious / depressed raw score (CBCL^5^) | Score ranging from 0 to 26 | Parent |
|  |  | Child withdrawn / depressed raw score (CBCL^5^) | Score ranging from 0 to 15 | Parent |
|  |  | Child somatic complains raw score (CBCL^5^) | Score ranging from 0 to 16 | Parent |
|  |  | Child social problems raw score (CBCL^5^) | Score ranging from 0 to 18 | Parent |
|  |  | Child thought problems raw score (CBCL^5^) | Score ranging from 0 to 18 | Parent |
|  |  | Child attention problems raw score (CBCL^5^) | Score ranging from 0 to 20 | Parent |
|  |  | Child rule breaking raw score (CBCL^5^) | Score ranging from 0 to 20 | Parent |
|  |  | Child aggressive behavior raw score (CBCL^5^) | Score ranging from 0 to 36 | Parent |
|  |  | Number of friends | Count | Youth |
|  |  | Number of close friends | Count | Youth |
|  | Physical health and attributes | Age at baseline | Months | Parent |
|  |  | Sex assigned at birth | 1=Male, 2=Female | Parent |
|  |  | Pubertal development scale (PDS^6^) category | 1=pre-pubertal, 2=early puberty, 3=mid-puberty, 4=late puberty, 5=post-pubertal | Parent |
|  |  | Number of days in the past 7 physically active | Days 0-7 | Youth |
|  |  | Number of school days in a week with physical education | Days 0-7 | Youth |
|  |  | Sleep Disturbance Scale for Children^7^: Disorders of Initiating and Maintaining Sleep (DIMS) | Score ranging from 7 to 35 | Parent |
|  |  | Sleep Disturbance Scale for Children^7^: Sleep Breathing disorders (SBD) | Score ranging from 3 to 15 | Parent |
|  |  | Sleep Disturbance Scale for Children^7^: Disorder of Arousal (DA) | Score ranging from 3 to 15 | Parent |
|  |  | Sleep Disturbance Scale for Children^7^: Sleep-Wake transition Disorders (SWTD) | Score ranging from 6 to 30 | Parent |
|  |  | Sleep Disturbance Scale for Children^7^: Disorders of Excessive Somnolence (DOES) | Score ranging from 5 to 25 | Parent |
|  |  | Sleep Disturbance Scale for Children^7^: Sleep Hyperhydrosis (SHY) | Score ranging from 2 to 10 | Parent |
|  |  | Sleep Disturbance Scale for Children^7^: Total Score on sleep disturbance scale for children | Score ranging from 26 to 126 | Parent |
| Environment | Home environment | Income-to-needs ratio (INR) | Based on US Department of Health and Human Services poverty thresholds (https://aspe.hhs.gov/topics/poverty-economic-mobility/poverty-guidelines/prior-hhs-poverty-guidelines-federal-register-references/2018-poverty-guidelines) | Parent |
|  |  | Family experiences: couldn't afford food | 1=Yes, 0=No | Parent |
|  |  | Family experiences: couldn't afford telephone | 1=Yes, 0=No | Parent |
|  |  | Family experiences: couldn't afford full rent or mortgage | 1=Yes, 0=No | Parent |
|  |  | Family experiences: evicted | 1=Yes, 0=No | Parent |
|  |  | Family experiences: utilities turned off | 1=Yes, 0=No | Parent |
|  |  | Family experiences: couldn't afford medical | 1=Yes, 0=No | Parent |
|  |  | Family experiences: couldn't afford dental | 1=Yes, 0=No | Parent |
|  |  | Family Environment Scale^8^: Conflict subscale score (parent-reported) | Score ranging from 0 to 9 | Parent |
|  |  | Family Environment Scale^8^: Conflict subscale score (youth-reported) | Score ranging from 0 to 9 | Youth |
|  |  | Parent behavior inventory^9^ - acceptance subscale (for responding parent) | Score ranging from 1 to 3 | Youth |
|  |  | Parent behavior inventory^9^ - acceptance subscale (for other parent) | Score ranging from 1 to 3 | Youth |
|  |  | Parental monitoring score^10^ | Score ranging from 1 to 5 | Youth |
|  |  | Lax parent rules on substances | Score ranging from 0 to 15 | Parent |
|  |  | Parent rules on substances not set | 1=Yes, 0=No |  |
|  | Traumatic events – from the K-SADS^11^ post-traumatic stress disorder module | Car accident | 1=Yes, 0=No | Parent |
|  |  | Other accident | 1=Yes, 0=No | Parent |
|  |  | Witnessed or affected by a fire | 1=Yes, 0=No | Parent |
|  |  | Witnessed or affected by natural disaster | 1=Yes, 0=No | Parent |
|  |  | Witnessed or affected by terrorist attack | 1=Yes, 0=No | Parent |
|  |  | War zone | 1=Yes, 0=No | Parent |
|  |  | Witnessed someone shot/stabbed | 1=Yes, 0=No | Parent |
|  |  | Shot/stabbed/beaten by a family member | 1=Yes, 0=No | Parent |
|  |  | Shot/stabbed/beaten by someone in the home | 1=Yes, 0=No | Parent |
|  |  | Beaten to the point of bruises at home | 1=Yes, 0=No | Parent |
|  |  | Threatened to be killed by non-family member | 1=Yes, 0=No | Parent |
|  |  | Threatened to be killed by family member | 1=Yes, 0=No | Parent |
|  |  | Witnessed violence in the home | 1=Yes, 0=No | Parent |
|  |  | Grown up in a home touched privates inappropriately | 1=Yes, 0=No | Parent |
|  |  | Other adult touched privates inappropriately | 1=Yes, 0=No | Parent |
|  |  | Peer forced sexual activity | 1=Yes, 0=No | Parent |
|  |  | Bullied | 1=Yes, 0=No | Parent |
|  |  | Death of a loved one | 1=Yes, 0=No | Parent |
|  |  | Any accident | 1=Yes, 0=No | Parent |
|  |  | Witnessed violence | 1=Yes, 0=No | Parent |
|  |  | Threat of harm/death | 1=Yes, 0=No | Parent |
|  |  | Physical abuse | 1=Yes, 0=No | Parent |
|  |  | Sexual abuse | 1=Yes, 0=No | Parent |
|  | Neighborhood environment | SRPF^12^ school environment total score | Score ranging from 6 to 24 | Youth |
|  |  | Continuous peer deviance score | Score ranging from 0 to 32 | Youth |
|  |  | Any peer deviance | 1=Yes, 0=No | Youth |
|  |  | Community risk and protective factors | Score ranging from 0 to 21 | Parent |
|  |  | Neighborhood safety and crime (survey)^13^ | Score ranging from 1 to 5 | Census |
|  |  | Area Deprivation Index (ADI)^14^ | Score ranging from 1 to 100 | Census |
|  |  | Child Opportunity Index (COI^15^ v2.0): |  |  |
|  |  | Adult educational attainment | Score ranging from 1 to 100 | Census |
|  |  | Advanced placement course enrollment | Score ranging from 1 to 100 | Census |
|  |  | College enrollment in nearby institutions | Score ranging from 1 to 100 | Census |
|  |  | Early childhood education centers | Score ranging from 1 to 100 | Census |
|  |  | Early childhood education enrollment | Score ranging from 1 to 100 | Census |
|  |  | High school graduation rate | Score ranging from 1 to 100 | Census |
|  |  | High quality early childhood education centers | Score ranging from 1 to 100 | Census |
|  |  | School poverty | Score ranging from 1 to 100 | Census |
|  |  | Teacher experience | Score ranging from 1 to 100 | Census |
|  |  | Third grade math proficiency | Score ranging from 1 to 100 | Census |
|  |  | Third grade reading proficiency | Score ranging from 1 to 100 | Census |
|  |  | Access to green space | Score ranging from 1 to 100 | Census |
|  |  | Access to healthy food | Score ranging from 1 to 100 | Census |
|  |  | Airborne microparticles | Score ranging from 1 to 100 | Census |
|  |  | Extreme heat exposure (days above 90 in a year) | Score ranging from 1 to 100 | Census |
|  |  | Hazardous waste dump sites | Score ranging from 1 to 100 | Census |
|  |  | Health insurance coverage | Score ranging from 1 to 100 | Census |
|  |  | Housing vacancy rate | Score ranging from 1 to 100 | Census |
|  |  | Industrial pollutants in the air | Score ranging from 1 to 100 | Census |
|  |  | Ozone concentration | Score ranging from 1 to 100 | Census |
|  |  | Walkability | Score ranging from 1 to 100 | Census |
|  |  | Commute duration | Score ranging from 1 to 100 | Census |
|  |  | Employment rate | Score ranging from 1 to 100 | Census |
|  |  | High-skill employment | Score ranging from 1 to 100 | Census |
|  |  | Home ownership rate | Score ranging from 1 to 100 | Census |
|  |  | Median household income | Score ranging from 1 to 100 | Census |
|  |  | Poverty rate | Score ranging from 1 to 100 | Census |
|  |  | Public assistance rate | Score ranging from 1 to 100 | Census |
|  |  | Single-headed households | Score ranging from 1 to 100 | Census |
|  |  | Gross residential density | Density at the census tract | Census |
|  |  | Census block with 2500 or more people | 1 = Urbanized Area; 2 = Urban Clusters ; 3 = Rural | Census |
|  |  | National Walkability Index | https://www.epa.gov/smartgrowth/national-walkability-index-user-guide-and-methodology | Census |
|  |  | Average Annual Daily Traffic | Count | Census |
|  |  | Proximity to roads | Meters | Census |
|  |  | Social Vulnerability Index^16^ | Score ranging from 0 to 1 | Census |
|  |  | ICPSR Unified Crime Report: adult violent crimes in the county, per SD | Count in census tract | Census |
|  |  | Estimated lead risk in census tract | Percentage of individuals below -125 percent of poverty level in census tract of primary residential address | Census |
|  |  | Total monthly average night light radiance | nW/cm2/sr | Census |
|  |  | NonWhite-White Dissimilarity Index, metro level^17-19^ | 0 (proportional distribution of racial groups) to 1 (complete segregation) | Census |
|  |  | NonWhite-White Interaction or Exposure Index, metro level^17-19^ | 0 (proportional distribution of racial groups) to 1 (complete segregation) |  |
|  |  | Index of Concentration at the Extremes (Income)^20^ | Score ranging from -1 (concentrated disadvantage) to 1 (concentrated privilege) | Census |
|  |  | Index of Concentration at the Extremes (Income + Race)^20^ | Score ranging from -1 (concentrated disadvantage) to 1 (concentrated privilege) | Census |
|  |  | Index of Concentration at the Extremes (Race)^20^ | Score ranging from -1 (concentrated disadvantage) to 1 (concentrated privilege) | Census |
|  |  | State-level indicators of racism (survey)^21^ | Higher scores indicate greater stigma | ABCD |
|  |  | State-level indicators of sexual orientation bias (survey)^21^ | Higher scores indicate greater stigma | ABCD |
|  |  | State-level indicators of sexism (survey)^21^ | Higher scores indicate greater stigma | ABCD |
|  |  | Medicaid expansion as of baseline year^22^ | 1=Yes, 0=No | State data |
| Family history | Parent demographics | Primary caregiver educational attainment | Years of education | Parent |
|  |  | Secondary caregiver educational attainment | Years of education | Parent |
|  |  | Parent married or living with a partner | 1=Yes, 0=No | Parent |
|  | Developmental history | Birthweight | Pounds | Parent |
|  |  | Breastfeeding | Months | Parent |
|  |  | Maternal age at child's birth | Years | Parent |
|  |  | Paternal age at child's birth | Years | Parent |
|  |  | Planned pregnancy | 1=Yes, 0=No | Parent |
|  |  | Substance use during pregnancy | 1=Yes, 0=No | Parent |
|  |  | Prenatal vitamin use | 1=Yes, 0=No | Parent |
|  |  | Caffeine use during pregnancy | 1=Yes, 0=No | Parent |
|  |  | Birth complications | 1=Yes, 0=No | Parent |
|  |  | Birth by Caesarian section | 1=Yes, 0=No | Parent |
|  | Parent mental health | Father alcohol problem | 1=Yes, 0=No | Parent |
|  |  | Mother alcohol problem | 1=Yes, 0=No | Parent |
|  |  | Mother drug problem | 1=Yes, 0=No | Parent |
|  |  | Father drug problem | 1=Yes, 0=No | Parent |
|  |  | Parent drug use for non-medicinal purposes (ASR^23^) | Standardized score | Parent |
|  |  | Parent I drink too much alcohol or get drunk (ASR^23^) | Standardized score | Parent |
|  |  | Parent days in last 6 months using drugs (ASR^23^) | Standardized score | Parent |
|  |  | Parent anxious/depressed t-score (ASR^23^) | Standardized score | Parent |
|  |  | Parent withdrawn t-score (ASR^23^) | Standardized score | Parent |
|  |  | Parent somatic complains t-score (ASR^23^) | Standardized score | Parent |
|  |  | Parent thought problems t-score (ASR^23^) | Standardized score | Parent |
|  |  | Parent attention problems t-score (ASR^23^) | Standardized score | Parent |
|  |  | Parent aggressive behavior t-score (ASR^23^) | Standardized score | Parent |
|  |  | Parent rule-breaking t-score (ASR^23^) | Standardized score | Parent |
|  |  | Parent intrusive t-score (ASR^23^) | Standardized score | Parent |
|  |  | Father seen a mental health professional | 1=Yes, 0=No | Parent |
|  |  | Mother seen a mental health professional | 1=Yes, 0=No | Parent |
|  |  | Father hospitalized for emotional/mental health problem | 1=Yes, 0=No | Parent |
|  |  | Mother hospitalized for emotional/mental health problem | 1=Yes, 0=No | Parent |

1. Carver CS, White TL. Behavioral Inhibition, Behavioral Activation, and Affective Responses to Impending Reward and Punishment: The BIS/BAS Scales. *Journal of personality and social psychology*. 1994;67(2):319-333. doi:10.1037/0022-3514.67.2.319

2. Watts AL, Smith GT, Barch DM, Sher KJ. Factor structure, measurement and structural invariance, and external validity of an abbreviated youth version of the UPPS-P Impulsive Behavior Scale. *Psychol Assess*. Apr 2020;32(4):336-347. doi:10.1037/pas0000791

3. Goodman R. The Strengths and Difficulties Questionnaire: A Research Note. *Journal of child psychology and psychiatry*. 1997;38(5):581-586. doi:10.1111/j.1469-7610.1997.tb01545.x

4. Goodman R, Meltzer H, Bailey. The strengths and difficulties questionnaire: A pilot study on the validity of the self-report version. *European child & adolescent psychiatry*. 1998;7(3):125-130. doi:10.1007/s007870050057

5. Achenbach TM. Manual for the Child Behavior Checklist/4-18 and 1991 profile. *University of Vermont, Department of Psychiatry*. 1991;

6. Kanwar P. Pubertal development and problem behaviours in Indian adolescents. *International journal of adolescence and youth*. 2020;25(1):753-764. doi:10.1080/02673843.2020.1739089

7. Bruni O, Ottaviano S, Guidetti, et al. The sleep disturbance scale for children (SDSC) construction and validation of an instrument to evaluate sleep disturbances in childhood and adolescence. *Journal of sleep research*. 1996;5(4):251-261. doi:10.1111/j.1365-2869.1996.00251.x

8. Moos RH, Moos BS. Family environment scale manual: Development, applications, research. *(No Title)*. 1994;

9. Schaefer ES. Children's reports of parental behavior: An inventory. *Child development*. 1965:413-424.

10. Karoly HC, Callahan T, Schmiege SJ, Feldstein Ewing SW. Evaluating the Hispanic paradox in the context of adolescent risky sexual behavior: The role of parent monitoring. *Journal of pediatric psychology*. 2016;41(4):429-440.

11. Geller B, Zimerman B, Williams M, et al. Reliability of the Washington University in St. Louis Kiddie Schedule for Affective Disorders and Schizophrenia (WASH-U-KSADS) mania and rapid cycling sections. *Journal of the American Academy of Child & Adolescent Psychiatry*. 2001;40(4):450-455.

12. Arthur MW, Briney JS, Hawkins JD, Abbott RD, Brooke-Weiss BL, Catalano RF. Measuring risk and protection in communities using the Communities That Care Youth Survey. *Evaluation and program planning*. 2007;30(2):197-211.

13. Echeverria SE, Diez-Roux AV, Link BG. Reliability of self-reported neighborhood characteristics. *Journal of Urban Health*. 2004;81(4):682-701.

14. Kind AJH, Jencks S, Brock J, et al. Neighborhood socioeconomic disadvantage and 30-day rehospitalization: a retrospective cohort study. *Annals of internal medicine*. 2014;161(11):765-774. doi:10.7326/M13-2946

15. Noelke C. *The Geography of Child Opportunity: Why Neighborhoods Matter For Equity*. Institute for Child, Youth & Family Policy (ICYFP), Heller School For Social …; 2020.

16. Flanagan BE, Gregory EW, Hallisey EJ, Heitgerd JL, Lewis B. A social vulnerability index for disaster management. *Journal of homeland security and emergency management*. 2011;8(1):0000102202154773551792.

17. Duncan OD, Duncan B. A Methodological Analysis of Segregation Indexes. *American sociological review*. 1955;20(2):210-217. doi:10.2307/2088328

18. Massey DS, Denton NA. The Dimensions of Residential Segregation*. *Social Forces*. 1988;67(2):281-315. doi:10.1093/sf/67.2.281

19. White MJ. Segregation and Diversity Measures in Population Distribution. *Population Index*. 1986;52(2):198-221. doi:10.2307/3644339

20. Krieger N, Kim R, Feldman J, Waterman PD. Using the Index of Concentration at the Extremes at multiple geographical levels to monitor health inequities in an era of growing spatial social polarization: Massachusetts, USA (2010–14). *International Journal of Epidemiology*. 2018;47(3):788-819. doi:10.1093/ije/dyy004

21. Hatzenbuehler ML, Weissman DG, McKetta S, et al. Smaller Hippocampal Volume Among Black and Latinx Youth Living in High-Stigma Contexts. *Journal of the American Academy of Child and Adolescent Psychiatry*. 2022;61(6):809-819. doi:10.1016/j.jaac.2021.08.017

22. <https://www.kff.org/affordable-care-act/state-indicator/state-activity-around-expanding-medicaid-under-the-affordable-care-act/?currentTimeframe=0&sortModel=%7B%22colId%22:%22Location%22,%22sort%22:%22asc%22%7D>

23. Achenbach TM, Verhulst F. Achenbach system of empirically based assessment (ASEBA). *Burlington, Vermont*. 2010;

### Table S2. Comparison of model discrimination using AUC by modeling strategy and outcome

|  | Fluid cognition | Crystallized cognition |
| --- | --- | --- |
| cv-xgboost | 0.6639 | 0.7191 |
| cv-SuperLearner | 0.6707 | 0.7116 |
| cv-LASSO | 0.6713 | 0.7544 |
| Logistic reg based on LASSO | 0.6781 | 0.7668 |
| cv = cross-validated using 10 mutually exclusive folds, retaining children from the same family in the same fold | | |

### Table S3. Odds ratios for retained predictors from models accounting for familial clustering

|  |  | **Fluid cognitive resilience** | | |  | **Crystallized cognitive resilience** | | |
| --- | --- | --- | --- | --- | --- | --- | --- | --- |
| **Child characteristics** |  | OR | 95% CI | *P* |  | OR | 95% CI | *P* |
| **Behavior and activities** | Participation in performing arts | 1.3 | (1.09,1.54) | .003 |  | 1.3 | (1.07,1.58) | .01 |
|  | Participation in visual arts |  |  |  |  | 1.33 | (1.05,1.69) | .02 |
| **Temperament** | Behavioral activation - drive | 0.97 | (0.95,1) | .02 |  | 0.99 | (0.96,1.02) | .41 |
|  | Positive urgency | 0.96 | (0.93,0.98) | .002 |  | 0.96 | (0.93,1) | .04 |
|  | Negative urgency |  |  |  |  | 0.96 | (0.92,1) | .04 |
|  | Lack of planning |  |  |  |  | 1.05 | (1,1.09) | .03 |
|  | Sensation-seeking |  |  |  |  | 1.06 | (1.02,1.1) | .001 |
|  | Prosocial behavior (youth report on self) |  |  |  |  | 0.7 | (0.54,0.9) | .007 |
| **Mental Health** | CBCL: Anxious / depressed |  |  |  |  | 1.08 | (1.04,1.12) | <.001 |
|  | CBCL: Social problems |  |  |  |  | 0.91 | (0.86,0.97) | .002 |
|  | CBCL: Attention problems | 0.93 | (0.91,0.95) | <.001 |  | 0.91 | (0.87,0.94) | <.001 |
|  | Number of close friends |  |  |  |  | 0.99 | (0.98,1) | .03 |
| **Physical health & attributes** | Age (months) | 1.02 | (1.01,1.03) | <.001 |  | 1.01 | (1,1.03) | .04 |
|  | Pubertal development score | 0.88 | (0.8,0.96) | .006 |  |  |  |  |
|  | Physical activity | 1.04 | (1.01,1.08) | .01 |  | 1.07 | (1.02,1.11) | .002 |
| **Family history** |  |  |  |  |  |  |  |  |
| **Demographics** | Primary caregiver educational attainment | 1.05 | (1,1.11) | .03 |  | 1.04 | (0.98,1.1) | .15 |
|  | Secondary caregiver educational attainment | 1.02 | (0.98,1.06) | .35 |  | 1.09 | (1.04,1.14) | <.001 |
| **Developmental history** | Birthweight | 1.12 | (1.06,1.19) | <.001 |  | 1.17 | (1.09,1.25) | <.001 |
|  | Breastfeeding (months) | 1.01 | (1,1.02) | 0.08 |  | 1.01 | (1,1.02) | .07 |
|  | Parent attention problems |  |  |  |  | 1.04 | (1.02,1.06) | <.001 |
|  | Mother seen a mental health professional |  |  |  |  | 1.19 | (0.97,1.47) | .10 |
| **Environment** |  |  |  |  |  |  |  |  |
| **Home environment** | Income-to-needs | 1.17 | (1,1.36) | .05 |  | 1.55 | (1.3,1.85) | <.001 |
|  | Family conflict (youth-reported) |  |  |  |  | 0.93 | (0.89,0.98) | .006 |
|  | Lax parental rules on substances | 0.91 | (0.85,0.97) | .003 |  |  |  |  |
| **Neighborhood** | School environment |  |  |  |  | 0.96 | (0.93,0.99) | .02 |
|  | Neighborhood advanced placement enrollment |  |  |  |  | 2.06 | (1.34,3.16) | <.001 |
|  | Neighborhood school poverty |  |  |  |  | 1 | (0.99,1) | .63 |
|  | Neighborhood reading proficiency (3rd grade) |  |  |  |  | 1 | (1,1) | .55 |
|  | Neighborhood housing vacancy rate | 0.98 | (0.97,1) | .02 |  |  |  |  |
|  | Neighborhood industrial pollutants in the air |  |  |  |  | 1.06 | (1.02,1.1) | .001 |
|  | Neighborhood employment rate |  |  |  |  | 1.01 | (1,1.02) | .09 |
|  | Neighborhood poverty rate | 0.99 | (0.98,1) | .18 |  |  |  |  |
|  | Neighborhood public assistance rate | 1 | (0.98,1.01) | .44 |  | 1 | (0.99,1.01) | .69 |
|  | Index of Concentration at the Extremes (Income) | 0.99 | (0.49,1.98) | .98 |  | 1.12 | (0.53,2.34) | .77 |
|  | Index of Concentration at the Extremes (Income + Race) | 1.16 | (0.53,2.55) | .70 |  | 1.41 | (0.55,3.6) | .48 |

### Table S4a. Comparison of predictors selected as important for prediction of crystallized resilience in the low income-to-needs tertile and high income-to-needs tertile samples

|  |  | **Low income-to-needs tertile** | | **High income-to-needs tertile** | |
| --- | --- | --- | --- | --- | --- |
|  |  | **N=3373** | | **N=3805** | |
|  |  | **SHAP** | **Mean (SD)** | **SHAP** | **Mean (SD)** |
| **Child characteristics** |  |  |  |  |  |
| **Behaviour and activities** | Hours of screentime, weekdays |  |  | 0.06 | 1.98 (2.17) |
|  | Participation in performing arts | 0.04 | 0.38 (0.49) | 0.03 | 0.68 (0.47) |
|  | Participation in visual arts | **0.01** | **0.17 (0.38)** |  |  |
|  | Participation in other activities |  |  | 0.06 | 0.29 (0.45) |
| **Temperament** | Behavioral activation - drive | 0.01 | 4.63 (3.25) | 0.01 | 3.67 (2.83) |
|  | Behavioral activation - fun-seeking |  |  | 0.04 | 5.48 (2.51) |
|  | Positive urgency | 0.02 | 8.49 (3.12) | 0.03 | 7.53 (2.73) |
|  | Negative urgency | 0.02 | 8.73 (2.83) | 0.03 | 8.28 (2.45) |
|  | Lack of planning | 0.08 | 7.62 (2.56) | 0.01 | 7.83 (2.21) |
|  | Sensation-seeking | 0.01 | 9.56 (2.75) | 0.02 | 10.00 (2.61) |
|  | Prosocial behavior (youth report on self) | **0.01** | **1.67 (0.39)** |  |  |
| **Mental Health** | CBCL Anxious / depressed | 0.02 | 2.71 (3.27) | 0.04 | 2.35 (2.82) |
|  | CBCL Social problems | **0.02** | **2.12 (2.58)** |  |  |
|  | CBCL Attention problems | 0.11 | 3.45 (3.79) | 0.11 | 2.54 (3.10) |
|  | Number of friends |  |  | 0.02 | 20.91 (22.06) |
|  | Number of close friends | **0.01** | **6.63 (11.93)** |  |  |
| **Physical health & attributes** | Age (months) | 0.03 | 118.64 (7.45) | 0.07 | 119.28 (7.47) |
|  | Physical activity | **0.05** | **3.11 (2.37)** |  |  |
| **Family history** |  |  |  |  |  |
| **Demographics** | Primary caregiver educational attainment | 0.07 | 13.52 (2.25) | 0.01 | 17.11 (2.17) |
|  | Secondary caregiver educational attainment | 0.15 | 13.12 (2.62) | 0.09 | 16.69 (2.46) |
|  | Parent married or living with a partner |  |  | 0.02 | 0.92 (0.27) |
| **Developmental history** | Birthweight | 0.09 | 6.93 (1.44) | 0.05 | 7.05 (1.47) |
|  | Planned pregnancy |  |  | 0.05 | 0.81 (0.40) |
|  | Breastfeeding (months) | 0.06 | 5.94 (7.80) | 0.15 | 9.00 (7.83) |
| **Parent mental health** | Parent attention problems | **0.07** | **54.55 (6.65)** |  |  |
|  | Mother seen a mental health professional | 0.02 | 0.31 (0.46) | 0.01 | 0.34 (0.47) |
| **Environment** |  |  |  |  |  |
| **Home environment** | Income-to-needs | 0.17 | 1.01 (0.60) | 0.04 | 6.36 (1.15) |
|  | Family conflict (youth-reported) | **0.04** | **2.44 (2.06)** |  |  |
|  | Parental monitoring score |  |  | 0.03 | 4.46 (0.44) |
| **Neighborhood** | School environment | 0.03 | 19.96 (3.08) | 0.01 | 19.96 (2.58) |
|  | Area Deprivation Index |  |  | 0.05 | 24.60 (18.19) |
|  | Neighborhood advanced placement enrollment | **0.06** | **0.41 (0.23)** |  |  |
|  | Neighborhood college enrollment |  |  | 0.02 | 46.80 (10.11) |
|  | Neighborhood early childhood education enrollment |  |  | 0.02 | 59.85 (24.44) |
|  | Neighborhood high quality early childhood education centers |  |  | 0.01 | 0.39 (4.43) |
|  | Neighborhood school poverty | **0.03** | **64.44 (24.68)** |  |  |
|  | Neighborhood reading proficiency (3rd grade) | 0.01 | 183.17 (65.38) | 0.03 | 243.10 (63.98) |
|  | Neighborhood access to green space |  |  | 0.02 | 27.86 (19.65) |
|  | Neighborhood industrial pollutants in the air | **0.03** | **6.83 (2.81)** |  |  |
|  | Neighborhood employment rate | **0.06** | **74.67 (10.71)** |  |  |
|  | Neighborhood high-skill employment |  |  | 0.04 | 51.22 (14.02) |
|  | Neighborhood public assistance rate | **0.03** | **20.15 (14.88)** |  |  |
|  | Adult violent crimes |  |  | 0.02 | 3377.31 (7625.24) |
|  | Index of Concentration at the Extremes (Income) | **0.05** | **-0.04 (0.28)** |  |  |
|  | Index of Concentration at the Extremes (Income + Race) | **0.13** | **0.05 (0.24)** |  |  |

Bolded cells indicate factors unique to the low-income group analysis. Text in green indicates factors common across the low-income and high-income analyses.

### Table S4b. Comparison of predictors selected as important for prediction of fluid resilience in the low income-to-needs tertile and high income-to-needs tertile samples

|  |  | **Low income-to-needs tertile** | | **High income-to-needs tertile** | |
| --- | --- | --- | --- | --- | --- |
|  |  | **N=3373** | | **N=3805** | |
|  |  | **SHAP** | **Mean (SD)** | **SHAP** | **Mean (SD)** |
| **Child characteristics** |  |  |  |  |  |
| Behavior and activities | Hours of screentime, weekdays |  |  | 0.01 | 1.98 (2.17) |
|  | Participation in performing arts | 0.06 | 0.38 (0.49) | 0.04 | 0.68 (0.47) |
| Temperament | Behavioral activation - drive | 0.04 | 4.63 (3.25) | 0.03 | 3.67 (2.83) |
|  | Positive urgency | **0.08** | **8.49 (3.12)** |  |  |
|  | Lack of planning |  |  | 0.01 | 7.83 (2.21) |
|  | Lack of perseverance |  |  | 0.08 | 6.89 (2.09) |
| Mental Health | CBCL Attention problems | 0.16 | 3.45 (3.79) | 0.18 | 2.54 (3.10) |
| Physical health & attributes | Age (months) | 0.08 | 118.64 (7.45) | 0.07 | 119.28 (7.47) |
|  | Female biological sex |  |  | 0.04 | 1.48 (0.50) |
|  | Pubertal development score | **0.04** | **1.94 (0.92)** |  |  |
|  | Physical activity | **0.01** | **3.11 (2.37)** |  |  |
| **Family history** |  |  |  |  |  |
| Demographics | Primary caregiver educational attainment | 0.03 | 13.52 (2.25) | 0.01 | 17.11 (2.17) |
|  | Secondary caregiver educational attainment | 0.03 | 13.12 (2.62) | 0.06 | 16.69 (2.46) |
| Developmental history | Birthweight | 0.09 | 6.93 (1.44) | 0.07 | 7.05 (1.47) |
|  | Planned pregnancy |  |  | 0.02 | 0.81 (0.40) |
|  | Breastfeeding (months) | 0.09 | 5.94 (7.80) | 0.06 | 9.00 (7.83) |
| **Environment** |  |  |  |  |  |
| Home environment | Income-to-needs | **0.07** | **1.01 (0.60)** |  |  |
|  | Lax parental rules on substances | **0.02** | **2.65 (1.30)** |  |  |
| Neighborhood | Neighborhood advanced placement enrollment |  |  | 0.04 | 0.50 (0.22) |
|  | Neighborhood health insurance coverage |  |  | 0.04 | 93.18 (5.22) |
|  | Neighborhood housing vacancy rate | **0.06** | **9.08 (7.42)** |  |  |
|  | Neighborhood high-skill employment |  |  | 0.03 | 51.22 (14.02) |
|  | Neighborhood poverty rate | **0.03** | **19.87 (13.40)** |  |  |
|  | Neighborhood public assistance rate | **0.05** | **20.15 (14.88)** |  |  |
|  | National Walkability Index |  |  | 0.02 | 10.10 (4.04) |
|  | NonWhite-White Interaction or Exposure Index, metro-level |  |  | 0.04 | 0.49 (0.17) |
|  | Index of Concentration at the Extremes (Income) | 0.11 | -0.04 (0.28) | 0.05 | 0.32 (0.23) |
|  | Index of Concentration at the Extremes (Income + Race) | **0.05** | **0.05 (0.24)** |  |  |
|  | State-level indicators of sexual orientation bias (survey) |  |  | 0.05 | -0.47 (0.82) |

Bolded cells indicate factors unique to the low-income group analysis. Text in green indicates factors common across the low-income and high-income analyses.

### Table S5a. Common predictors of crystallized cognitive performance in the bottom tertile and bottom quartile of the income-to-needs distribution

| Domain | Label | Predictor order (descending SHAP) in bottom tertile of income-to-needs | SHAP in the bottom tertile of income-to-needs | Predictor order (descending SHAP) in bottom quartile of income-to-needs | SHAP in the bottom quartile of income-to-needs |
| --- | --- | --- | --- | --- | --- |
| Culture and environment | Income-to-needs | 1 | 0.1657 | 1 | 0.1588 |
| Family history | Secondary caregiver educational attainment | 2 | 0.1459 | 2 | 0.1370 |
| Culture and environment | Index of Concentration at the Extremes (Income + Race) | 3 | 0.1306 | 8 | 0.0870 |
| Child characteristics | CBCL Attention problems | 4 | 0.1130 | 9 | 0.0806 |
| Family history | Birthweight | 5 | 0.0890 | 6 | 0.0999 |
| Child characteristics | Lack of planning | 6 | 0.0831 | -- | -- |
| Family history | Parent attention problems | 7 | 0.0709 | 11 | 0.0626 |
| Family history | Primary caregiver educational attainment | 8 | 0.0667 | 16 | 0.0235 |
| Family history | Breastfeeding (months) | 9 | 0.0638 | 4 | 0.1101 |
| Culture and environment | Neighborhood advanced placement enrollment | 10 | 0.0585 | 12 | 0.0561 |

### Table S5b. Common predictors of fluid cognitive performance in the bottom tertile and bottom quartile of the income-to-needs distribution

| Domain | Label | Predictor order (descending SHAP) in bottom tertile of income-to-needs | SHAP in the bottom tertile of income-to-needs | Predictor order (descending SHAP) in bottom tertile of income-to-needs | SHAP in the bottom quartile of income-to-needs |
| --- | --- | --- | --- | --- | --- |
| Child characteristics | CBCL Attention problems | 1 | 0.1608 | 1 | 0.1730 |
| Culture and environment | Index of Concentration at the Extremes (Income) | 2 | 0.1054 | -- | -- |
| Family history | Birthweight | 3 | 0.0921 | 3 | 0.0829 |
| Family history | Breastfeeding (months) | 4 | 0.0883 | 10 | 0.0474 |
| Child characteristics | Positive urgency | 5 | 0.0827 | 4 | 0.0770 |
| Child characteristics | Age (months) | 6 | 0.0753 | 2 | 0.0947 |
| Culture and environment | Income-to-needs | 7 | 0.0687 | 5 | 0.0697 |
| Culture and environment | Neighborhood housing vacancy rate | 8 | 0.0642 | 6 | 0.0644 |
| Child characteristics | Participation in performing arts | 9 | 0.0587 | 20 | 0.0177 |
| Culture and environment | Index of Concentration at the Extremes (Income + Race) | 10 | 0.0513 | 17 | 0.0258 |

**References:**

1. Chen T, Guestrin C. XGBoost: A Scalable Tree Boosting System. In: *Proceedings of the 22nd ACM SIGKDD International Conference on Knowledge Discovery and Data Mining*. KDD ’16. Association for Computing Machinery; 2016:785-794. doi:10.1145/2939672.2939785

2. Laan MJ van der, Polley EC, Hubbard AE. Super Learner. *Stat Appl Genet Mol Biol*. 2007;6(1). doi:10.2202/1544-6115.1309

3. Zou H, Hastie T. Regularization and Variable Selection Via the Elastic Net. *J R Stat Soc Ser B Stat Methodol*. 2005;67(2):301-320. doi:10.1111/j.1467-9868.2005.00503.x

4. Wright MN, Ziegler A. ranger: A Fast Implementation of Random Forests for High Dimensional Data in C++ and R. *J Stat Softw*. 2017;77:1-17. doi:10.18637/jss.v077.i01

5. Kooperberg C, Bose S, Stone CJ. Polychotomous Regression. *J Am Stat Assoc*. 1997;92(437):117-127. doi:10.2307/2291455

6. Lundberg S, Lee SI. A Unified Approach to Interpreting Model Predictions. Published online November 24, 2017. doi:10.48550/arXiv.1705.07874

7. Rakesh D, Zalesky A, Whittle S. Assessment of Parent Income and Education, Neighborhood Disadvantage, and Child Brain Structure. *JAMA Netw Open*. 2022;5(8):e2226208-e2226208. doi:10.1001/JAMANETWORKOPEN.2022.26208

8. Taylor RL, Cooper SR, Jackson JJ, Barch DM. Assessment of Neighborhood Poverty, Cognitive Function, and Prefrontal and Hippocampal Volumes in Children. *JAMA Netw Open*. 2020;3(11):e2023774. doi:10.1001/jamanetworkopen.2020.23774
